# Natural Allelic Variations in *IbCHYR1*–*IbZnFR* Complex Regulate *Fusarium* Root Rot Resistance in Sweet Potato

**DOI:** 10.1002/advs.202415202

**Published:** 2025-06-26

**Authors:** Huan Zhang, Zhuoru Dai, Xiaochen Zhang, Meiqi Shang, Xiaoru Gao, Ruiqi Ma, Limeng Zhao, Xiaoli Zhang, Qingchang Liu, Hong Zhai, Shaopei Gao, Ning Zhao, Qinghe Cao, Qiang Li, Shaozhen He

**Affiliations:** ^1^ Key Laboratory of Sweet Potato Biology and Biotechnology Ministry of Agriculture/Beijing Key Laboratory of Crop Genetic Improvement/Laboratory of Crop Heterosis and Utilization Ministry of Education College of Agronomy & Biotechnology China Agricultural University Beijing 10093 China; ^2^ Sanya Institute of China Agricultural University Sanya 572025 China; ^3^ Xuzhou Institute of Agricultural Sciences in Jiangsu Xuhuai District Xuzhou 221131 China; ^4^ Beijing Plant Protection Station Beijing 100029 China

**Keywords:** *FfRlpA2*, *Fusarium* root rot, *IbCHYR1*, *IbZnFR*, sweet potato

## Abstract

Sweet potato (*Ipomoea batatas*) is a globally important autohexaploid root and tuber crop. *Fusarium* root rot threatens its entire growth, harvest, and storage period, thereby reducing yield and quality. Therefore, a deeper understanding of *Fusarium* pathogenicity and sweet potato defense is urgently required. Here, two single nucleotide polymorphisms are identified within the promoter region of the *I. batatas* CHY zinc‐finger and ring protein1 (*IbCHYR1*) gene that encode an E3 ubiquitin ligase linked to root rot resistance. In susceptible varieties, the high dosage allele *Pro*::*IbCHYR1^Hap1^* leads to increased expression of *IbCHYR1*. Overexpression of *IbCHYR1* increases susceptibility to root rot and *Fusarium* wilt. *IbCHYR1* interacts with the *I. batatas* CCCH‐type zinc‐finger protein (*IbZnFR*) to promote its degradation. *IbZnFR* confers resistance to root rot and *Fusarium* wilt and improves yield by more than 10%. The high dosage *Pro*::*IbZnFR^Hap2^* allele is associated with resistance to root rot disease. Moreover, *FfRlpA2*, a conserved *Fusarium* effector, is identified as a protease inhibitor that stabilizes and hijacks *IbCHYR1* to degrade *IbZnFR*, thereby inhibiting multiple defense pathways. These findings provide insights into *Fusarium* pathogenicity and a genetic basis for root rot research and improvement of disease‐resistant sweet potato varieties.

## Graphical abstract

FfRlpA2, a conserved Fusarium effector, functions as a protease inhibitor by hijacking the E3 ubiquitin ligase IbCHYR1 to degrade the resistance factor IbZnFR, thereby promoting Fusarium pathogenicity. Conversely, the high dosage Pro::IbZnFR^Hap2^ allele was associated with resistance to root rot disease. IbZnFR conferred resistance to both root rot and Fusarium wilt and improved yield by more than 10%.

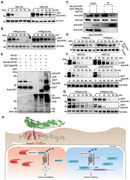

## 1 Introduction

Sweet potato, *Ipomoea batatas* [L.] Lam. (2*n* = B_1_B_1_B_2_B_2_B_2_B_2_ = 6*x* = 90), holds significant economic importance as a root and tuber crop, ranking eighth globally with a production volume of 86.4 million tons in 2022.^[^ ^1^ ^]^ Sweet potato is primarily grown by subsistence farmers in Asia and Africa, where they contribute to nutrition, serve as livestock feed, and play crucial roles in industrial and energy production.^[^ ^2^, ^3^ ^]^ The 2016 World Food Prize was awarded to the four scientists who revolutionized biofortification and improved human health using orange‐fleshed sweet potato.^[^ ^4^ ^]^ Despite its importance, sweet potato faces a formidable challenge from root rot, which causes a 30% loss in production, impacts quality, and occasionally results in complete crop failure.^[^ ^5^, ^6^ ^]^ This devastating soil‐borne disease also severely affects the production of many other economically important crops, such as cucumber, tomato, watermelon, strawberry, bean, beet, peanut cotton, and fruit trees. In China, ≈120 ha of main sweet‐potato‐producing regions are affected by root rot, leading to an annual economic loss exceeding 500 million yuan. This significantly impacts the farmers’ income. Root rot has also spread to Japan, where it caused an 8% reduction in sweet potato production in 2020 compared with that in the previous year, resulting in the lowest production level ever recorded (a decrease of 687 600 tons).^[^ ^7^ ^]^ As sweet potato is asexually propagated, infected tuberous roots can infect soil. Unfortunately, no effective chemical treatment is available. This not only hampers sweet potato farming in affected areas but also impedes the growth of sweet potato production enterprises.^[^ ^8^ ^]^ The primary strategy for mitigating root rot is to develop and cultivate resistant varieties. However, the key resistance genes and their underlying regulatory mechanisms, crucial for genetic engineering, remain inadequately explored.

*Fusarium* pathogens exhibit a biotrophic phase, during which they colonize the host. In the subsequent necrotrophic stage, these fungi employ strategies, such as producing carbohydrate‐active enzymes, cell‐wall‐degrading enzymes, and various effectors. These efforts prevent the thickening of host cell walls and hijack host secondary metabolic pathways, facilitating robust establishment and nutrient uptake from the host.^[^ ^9^ ^]^ *Fusarium* species are known globally as causal agents of significant crop diseases but present challenges owing to their high genetic variability and broad host specificity.^[^ ^10^ ^]^ These soil‐borne hemibiotrophic fungal pathogens cause root rot or wilting in more than 100 plant species.^[^ ^11^ ^]^ Despite their impact on sweet potato, the documentation of *Fusarium* root rot pathogens is limited. Two *Fusarium oxysporum* races were isolated from storage roots with root rot in Beijing, China, and *Fusarium solani* was identified as the cause of root rot in storage roots from Hebei Province, China.^[^ ^12^, ^13^ ^]^ In addition, *F. solani* CRI 24‐3 has been identified as the cause of root and stem rot in Guangdong, China.^[^ ^14^ ^]^ In NC, USA, six *Fusarium* species, namely *F. solani*, *F. oxysporum*, *Fusarium graminearum*, *Fusarium proliferatum*, *Fusarium acuminatum*, and *Fusarium incarnatum*, were identified as contributors to sweet potato root rot.^[^ ^5^ ^]^ In South Korea, root rot is predominantly caused by *F. oxysporum* and *F. solani*.^[^ ^6^, ^15^ ^]^ It has been reported that *F. oxysporum* also induces sweet potato *Fusarium* wilt, leading to yield losses from 10% to 50%.^[^ ^16^ ^]^ The genus *Fusarium* is notorious for producing secondary metabolites, a few of which are toxic to humans and animals.^[^ ^17^, ^18^, ^19^ ^]^ Consumption of moldy sweet potato can lead to diarrhea, abdominal pain, nausea, food poisoning, and acute gastroenteritis in humans.^[^ ^9^ ^]^ In cattle and other livestock, it can cause the cessation of rumination, muscle tremors, and difficulty in breathing.^[^ ^9^ ^]^ However, the specific pathogenic and control mechanisms underlying *Fusarium*‐mediated diseases in sweet potato remain unclear.

Pathogenic fungi deploy protein effectors in host cells to dampen the host immune responses.^[^ ^20^ ^]^ In response, plants recognize pathogen‐associated molecular patterns (PAMPs) to initiate pattern‐triggered immunity (PTI) and effector‐triggered immunity, which enables recognition of invading pathogens and activation of defense mechanisms.^[^ ^21^ ^]^ Recent studies have revealed pathogen–host interaction mechanisms. *Rhizoctonia solani* effector *RsRlpA* functions as a protea se inhibitor, suppressing the hypersensitive response in sugar beet to enhance virulence.^[^ ^22^ ^]^ *Magnaporthe oryzae* effector *MoRlpA* targets the rice cysteine protease *OsCathB*, inhibiting host cell death and promoting rice blast infection.^[^ ^23^ ^]^ In wheat, *TaFROG* competes with *F. graminearum* effector *Osp24* to protect the resistance protein *TaSnRK1α* from degradation, thereby enhancing fungal resistance.^[^ ^24^ ^]^ *M. oryzae* effector *AvrPi9* stabilizes the ubiquitin‐like protein *ANIP1* in rice, promoting the degradation of the defense regulator *OsWRKY62* to suppress host immunity.^[^ ^25^ ^]^

E3 ubiquitin ligases play critical roles in plant–pathogen interactions. *Verticillium dahliae* effector *VDAL* protects MYB6 from degradation by interacting with U‐box E3 ligases PUB25 and PUB26 to enhance *Verticillium* wilt resistance.^[^ ^26^ ^]^ *Phytophthora sojae* effector *Avr1d* acts as an E2 competitor, inhibiting the E3 ligase activity of soybean *GmPUB13* to facilitate infection.^[^ ^27^ ^]^ *M. oryzae* effector *AvrPiz‐t* targets the RING E3 Ligase APIP6 to suppress PAMP‐triggered immunity in rice.^[^ ^28^ ^]^ The F‐box E3 ligase OsFBK16 degrades OsPALs to negatively regulate rice blast resistance.^[^ ^29^ ^]^ CCCH‐type zinc‐finger proteins, characterized by motifs with three cysteines and one histidine (C─X_6–14_─C─X_4–5_─C─X_3_─H), are categorized into tandem and nontandem subtypes, which act as key regulators in plant disease resistance.^[^ ^30^ ^]^ In *Arabidopsis*, *AtC3H14* positively regulates resistance to *Botrytis cinerea* through the WRKY33‐dependent pathway.^[^ ^31^ ^]^ Overexpression of cotton *GhZFP1* in transgenic tobacco enhances resistance to *R. solani*.^[^ ^32^ ^]^ However, the molecular mechanisms by which E3 ubiquitin ligases interact with CCCH‐type zinc‐finger proteins to regulate plant immune responses remain poorly understood.

In this study, we found that the overexpression of the *I. batatas* CHY zinc‐finger and ring protein1 (*IbCHYR1*) gene, which encodes an E3 ubiquitin ligase, leads to susceptibility to root rot and *Fusarium* wilt. In susceptible varieties, the high dosage allele *Pro*::*IbCHYR1^Hap1^* resulted in the increased expression of *IbCHYR1*. A conserved protease inhibitor in *Fusarium*, *FfRlpA2*, hijacks *IbCHYR1* to degrade the CCCH‐type zinc‐finger protein, *IbZnFR*, via the 26S proteasome pathway. Notably, *IbZnFR* not only conferred resistance to both root rot and *Fusarium* wilt diseases through PTI but also contributed to an enhanced sweet potato yield. Our findings highlight the crucial role of natural variation and dosage of the *IbCHYR1*–*IbZnFR* complex in modulating the response to *Fusarium* through multiple signaling pathways in sweet potato.

## 2 Results

### 2.1 Root Rot Caused by *Fusarium foetens* Impairs the Entire Growth Cycle of Sweet Potato

**Figure 1:**
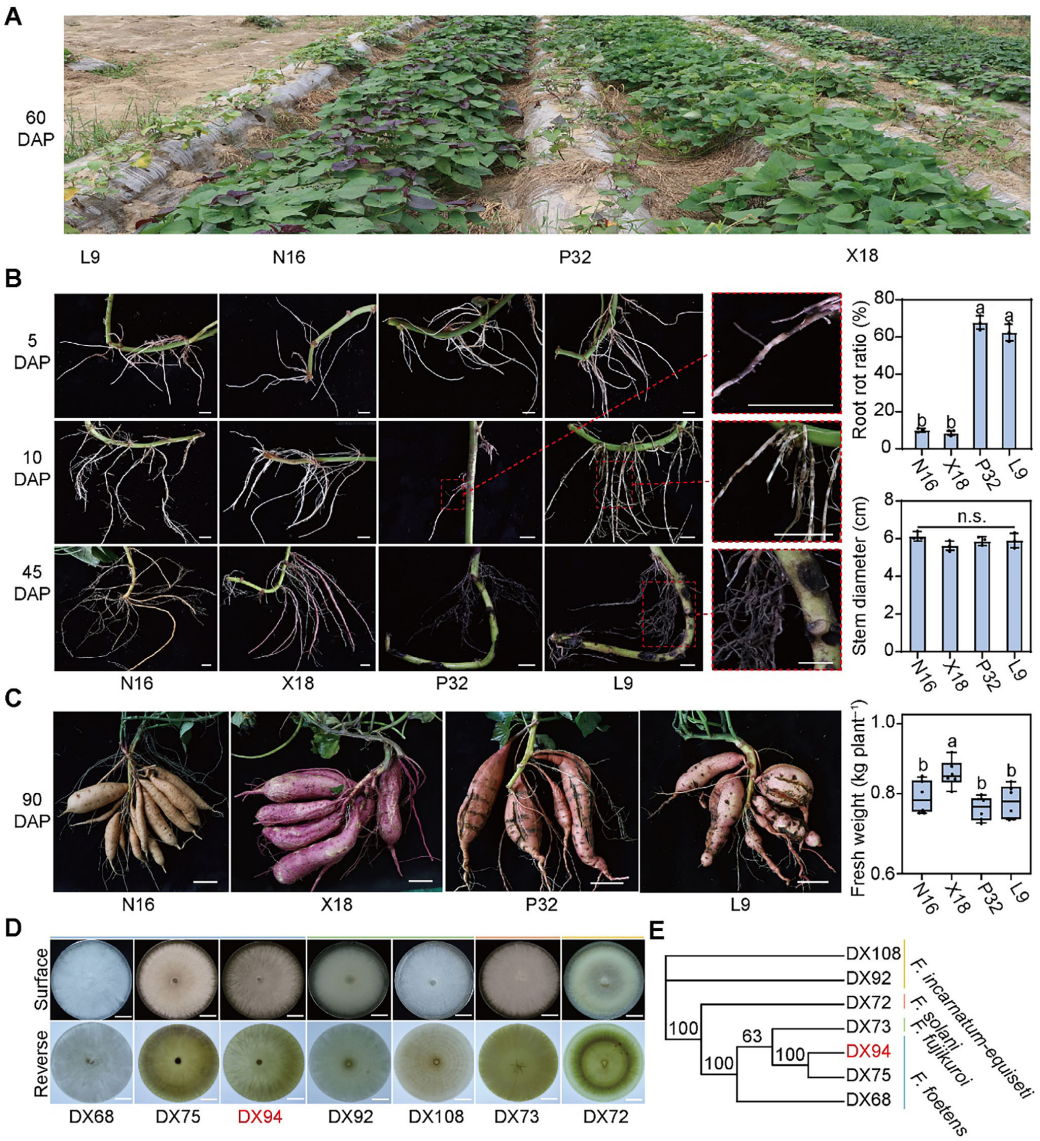
The entire growth period of sweet potato is threatened by root rot disease. A) Two root rot resistant (X18 and N16) and two susceptible (L9 and P32) varieties are pictured in a severe root rot field in Daxing, Beijing 60 days after planting (DAP). X18, Xushu18; N16, Nongda16; L9, Longshu9; P32, Pushu32. B) Time‐course symptoms of root rot in a severe root rot field. Scale bars, 5 mm. Data are shown as the means ± standard deviation (S.D.) (*n* = 3). C) Susceptible varieties form storage roots with a dry dehiscent epidermis or black rot lesions in a mild root rot field. Scale bars, 7 cm. Data are shown as the means ± S.D. (*n* = 3). D) Colonies of seven fungal strains isolated and purified from diseased plants in Daxing, Beijing. Scale bars, 2 cm. E) Evolutionary tree constructed with the internal transcribed spacer, second largest subunit of RNA polymerase II, and translation elongation factor 1‐α sequences of seven different fungal strains. In (B) and (C), different letters indicate statistically significant differences (one‐way ANOVA followed by a post‐hoc Tukey test; *p* < 0.05).

We evaluated the root rot resistance of 157 sweet potato accessions and found that high‐value varieties such as Longshu9, Yanshu25, and Pushu32, with average yields of 40–75 t ha^−1^, were highly susceptible. To gain a comprehensive understanding of root rot, we observed the typical symptoms of two resistant (Xushu18 and Nongda16) and two susceptible (Longshu9 and Pushu32) varieties throughout their growth cycles in severe and mild root‐rot fields in Daxing, Beijing, where root rot is the most severe in China (**Figures** **1** and S1 (Supporting Information)). In fields with severe root rot, the fibrous roots of susceptible varieties show black spots that gradually expand to cause root necrosis and aboveground wilting, resulting in no yield, whereas resistant varieties exhibit normal plant growth and can form storage roots (Figure 1A,B and Figure S1 (Supporting Information)). In fields with mild root rot, the storage roots of susceptible varieties with dry dehiscent epidermis and black rot lesions were toxic to humans and germinated slowly (Figure 1C). These results indicated that root rot poses a significant threat to the yield and quality of sweet potato.

To identify the pathogenic fungi that cause root rot in Daxing, Beijing, we isolated and purified seven fungal strains from diseased plants (Figure 1D). These strains belonged to four *Fusarium* species: three strains belonged to *F. foetens* (sister taxon of the *F. oxysporum* species complex),^[^ ^33^ ^]^ two strains belonged to *F. solani*, and one strain each belonged to *Fusarium fujikuroi* and *Fusarium incarnatum‐equiseti* (Figure 1E). Pathogenicity tests revealed that *F. foetens* strain DX94 had the highest level of pathogenicity (Figure S2, Supporting Information).

### 2.2 High Dosage *IbCHYR1Hap1* Allele Suppresses Resistance to Root Rot and *Fusarium* Wilt

**Figure 2:**
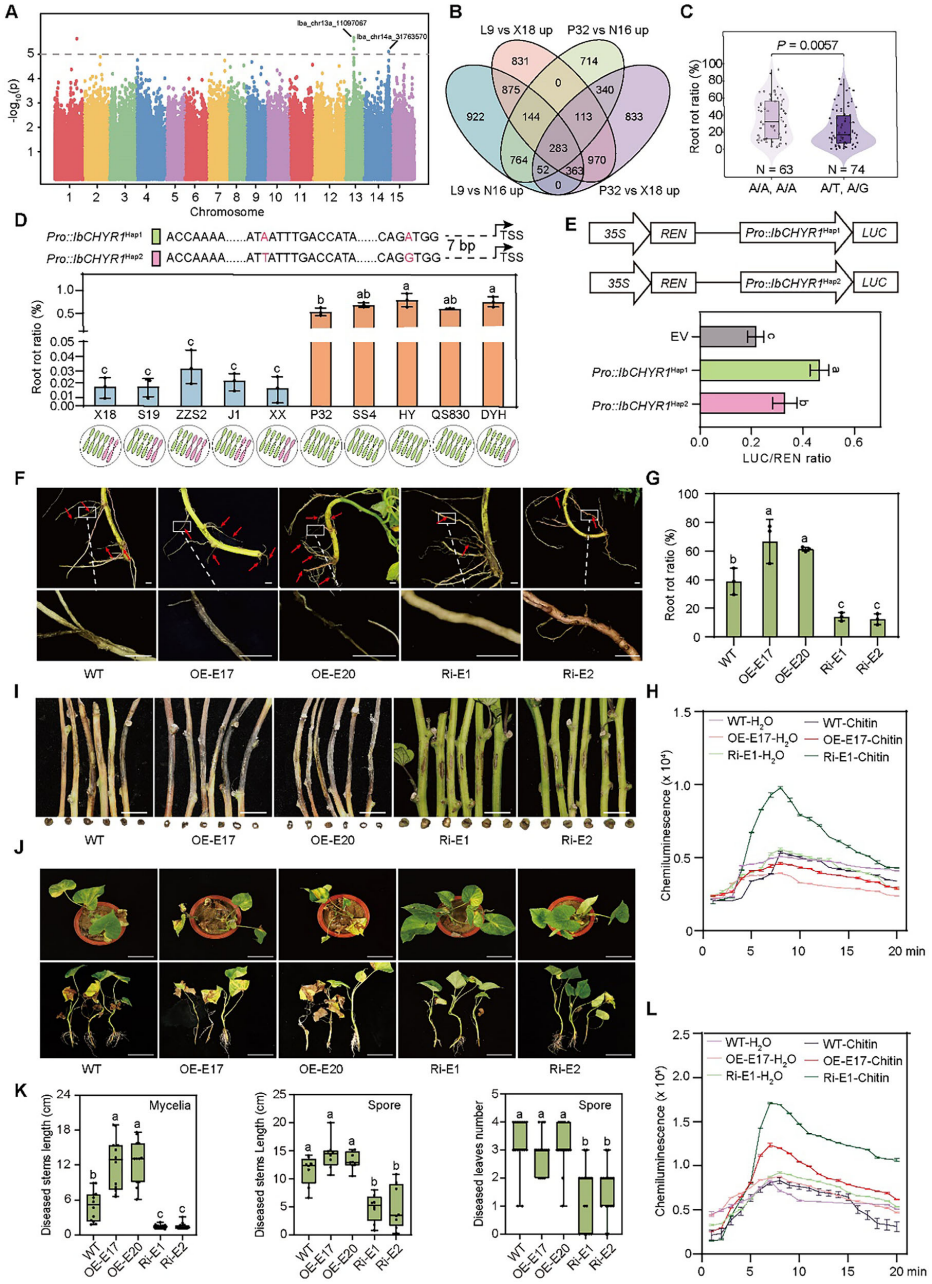
High dosage of the *IbCHYR1^Hap1^* allele suppresses sweet potato resistance to root rot and *Fusarium* wilt. A) Manhattan plot of a GWAS for root rot resistance performed on 157 sweet potato accessions. The horizontal dashed line indicates the significance threshold of GWAS (−log_10_[*p*] = 5.0). The lead SNPs are shown with their location information. B) In total, 283 differentially expressed genes, including *IbCHYR1*, upregulated in the roots of two susceptible varieties but downregulated in resistant ones by RNA‐seq at 10 days after planting in a root rot field. C) Violin plots depicting the root rot ratio of sweet potato accessions harboring the indicated SNP types at position *Iba_chr14a_31593974* and *Iba_chr14a_31594027* within the promoter region of *IbCHYR1*. D) The root rot ratio, and genotype and dosage of *IbCHYR1* of resistant and susceptible sweet potato varieties. Data are shown as the means ± S.D. (*n* = 3). X18, Xushu18; S19, Shangshu19; ZZS2, Zhanzishu2; J1, Ji10270; XX, Xinxiang; P32, Pushu32; SS4, Sushu4; HY, Hongyao; QS830, Quanshu830; DYH, Danyanhong. The red font indicates two SNPs (*Iba_chr14a_31593974* and *Iba_chr14a_31594027*) within the promoter region of *IbCHYR1*. cv., cultivar. TSS, transcription start site. E) Promoter activities of the *Pro::IbCHYR1^Hap1^* and *Pro::IbCHYR1^Hap2^* alleles. The empty vector was used as a negative control. The expression level of REN was used as an internal control. Data are shown as mean ± S.D. (*n* = 3). F,G) *IbCHYR1*‐OE lines formed more lesions than in the WT plants in contrast to that in the *IbCHYR1*‐Ri plants in the root rot field. Scale bars, 0.5 cm. Data are shown as the means ± S.D. (*n* = 3). H) ROS accumulation in the roots of *IbCHYR1* transgenic and WT plants in the root rot field. Data are shown as the means ± S.D. (*n* = 3). I,J) Development of disease symptoms in *IbCHYR1* transgenic and WT plants after *Fob* inoculation by mycelia (I, scale bars, 1 cm) or spore (J, scale bars, 10 cm). K) Statistical analysis of diseased leaves (*n* = 15) and stems (spore, *n* = 9; mycelia, *n* = 15) in *IbCHYR1*‐OE and *IbCHYR1*‐Ri transgenic plants and WT plants. Data are shown as the means ± S.D. L) ROS accumulation in the roots of *IbCHYR1* transgenic and WT plants at 0 DAI and 1 DAI by the spore infection method. Data are shown as the means ± S.D. (*n* = 15). In (D), (E), (G), and (K), different letters indicate statistically significant differences (one‐way ANOVA followed by a post‐hoc Tukey test; *p* < 0.05).

To identify the key factors regulating root rot resistance, a genome‐wide association study (GWAS) was conducted on 157 sweet potato accessions using a mixed linear model.^[^ ^34^ ^]^ Two significant loci (*Iba_chr13a_11097067* and *Iba_chr14a_31763570*; *p* < 1.0 × 10^−5^) associated with root rot resistance were identified, each containing 121 and 325 single nucleotide polymorphisms (SNPs) located within 0.53 and 0.55 Mb linkage disequilibrium (LD) blocks, encompassing 102 annotated protein‐coding genes (**Figures** **2**A and S3 and Table S2 (Supporting Information)). Further, the transcriptome sequencing (RNA‐seq) of roots 10 days after planting (DAP) in a root rot‐infested field revealed that of these 102 genes, two genes (*Iba_chr14aCG23010* and *Iba_chr14aCG23150*) were upregulated in the two susceptible varieties but downregulated in the resistant varieties (**Figure** **2**B and Table S3 (Supporting Information)). After excluding *Iba_chr14aCG23150* because of its extremely low expression levels, we focused on *Iba_chr14aCG23010*, which encodes a RING finger and CHY zinc‐finger domain‐containing protein (*IbCHYR1*; Figure S4 and Table S3, Supporting Information). Notably, two SNPs (*Iba_chr14a_31593974* and *Iba_chr14a_31594027*) were found within the promoter region of *IbCHYR1* (Figure 2C and Table S2 (Supporting Information)). Haplotype analysis revealed a significant reduction in the root rot ratio in heterozygous *IbCHYR1* individuals (represented as A/T, A/G) compared to homozygous ones (represented as A/A, A/A; Figure 2C). Subcellular fluorescence and fractionation assays revealed that *IbCHYR1* was localized to the nucleus, cell membrane, and cytoplasm (Figure S4C,D, Supporting Information).

As sweet potato is a highly heterozygous autohexaploid, we evaluated the relationship between the genotype and dosage of *IbCHYR1* across resistant and susceptible varieties using the high‐throughput tracking of mutations (Hi‐TOM) platform^[^ ^35^ ^]^ (Figure 2D). The genotypes of the two SNPs within the *IbCHYR1* promoter region were linked and divided into two major haplotypes (Figure 2D). The *Pro*::*IbCHYR1^Hap2^* allele was relatively more prevalent in resistant varieties (2–3 homologous chromosomes), whereas the *Pro*::*IbCHYR1^Hap1^* allele was more abundant in susceptible varieties (5–6 homologous chromosomes), reaching homozygosity (Figure 2D). Sequence analysis revealed that a bZIP binding motif (TTATT) was present in the *Pro*::*IbCHYR1^Hap1^* allele but was lost in the *Pro*::*IbCHYR1^Hap2^* allele (Figure S5A, Supporting Information).^[^ ^36^ ^]^ Dual‐luciferase (Dual‐LUC) reporter assays revealed higher promoter activity for the *Pro*::*IbCHYR1^Hap1^* allele than for the *Pro*::*IbCHYR1^Hap2^* allele (Figure 2E). Consistently, quantitative reverse transcription‐polymerase chain reaction (RT‐qPCR) analysis revealed the increased expression of *IbCHYR1^Hap1^* in susceptible Longshu9 roots, with or without DX94 infection, compared with that of *IbCHYR1^Hap2^* in resistant Xushu18 (Figure S4E, Supporting Information). These results suggest that the high‐dose haplotype *Pro*::*IbCHYR1^Hap1^* is associated with the susceptibility to root rot.

To investigate the role of *IbCHYR1* in host immunity, we cloned it from the highly expressing susceptible variety Longshu9 and generated overexpression and RNAi lines in the susceptible variety Lizixiang (Figure S6, Supporting Information). In vitro, *IbCHYR1*‐overexpressing (*IbCHYR1*‐OE) plants exhibited larger leaves, whereas *IbCHYR1*‐RNAi (*IbCHYR1*‐Ri) plants displayed smaller leaves than the wild‐type (WT) plants (Figure S7, Supporting Information). In contrast to field‐grown *IbCHYR1*‐Ri plants, field‐grown *IbCHYR1*‐OE plants exhibited significantly larger and thinner leaves and stems with thinner cell walls than in WT plants, (Figures S8 and S9A–C, Supporting Information). However, neither the overexpression nor the RNAi‐mediated silencing of *IbCHYR1* affected sweet potato yield (Figure S9D,E, Supporting Information).

Next, we evaluated the resistance of *IbCHYR1* transgenic lines to root rot. In contrast to the *IbCHYR1*‐Ri plants grown in the root‐rot‐infested field, the *IbCHYR1*‐OE lines grown in this filed exhibited more lesions, disrupted pith and cortex structures, lower cellulose and lignin contents, and a less pronounced reactive oxygen species (ROS) burst than in WT plants, (Figure 2F–H and Figures S10A,B, S11A, and S12A (Supporting Information)). Furthermore, the *IbCHYR1*‐OE and *IbCHYR1*‐Ri lines infected with the DX94 strain exhibited reduced and increased resistance, respectively, to this strain compared with that in the WT plants (Figure S13A, Supporting Information).

We then inoculated the lines with *F. oxysporum* f. sp *batatas* (*Fob*) and induced *Fusarium* wilt through mycelial and spore infection methods.^[^ ^16^ ^]^ After *Fob* infection, the *IbCHYR1*‐Ri lines exhibited fewer diseased leaves and stem necrotic regions, fewer disrupted pith and cortex structures, higher cellulose and lignin content, and a greater ROS burst than in the WT and *IbCHYR1*‐OE plants (Figure 2I–L and Figures S10C–E, S11C, and S12B (Supporting Information)). These findings suggest that *IbCHYR1* suppresses the resistance of sweet potato to root rot and *Fusarium* wilt.

### 2.3 *IbCHYR1* Interacts with *IbZnFR*

**Figure 3:**
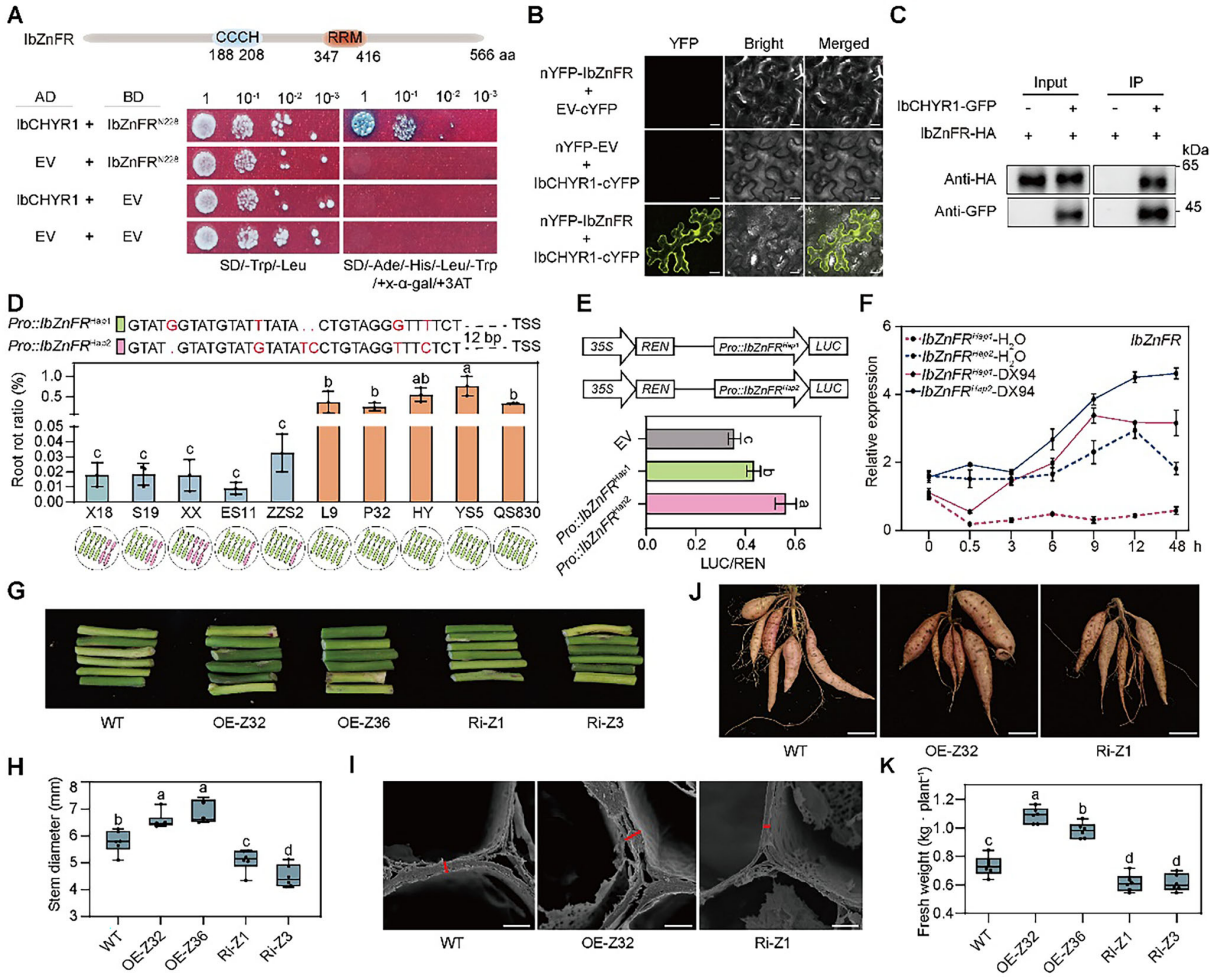
High dosage of the *Pro*::*IbZnFR^Hap2^* allele is associated with resistance to root rot disease. A) *IbCHYR1* interacts with *IbZnFR* in yeast. Yeast cells were plated onto SD/−Ade/−His/−Leu/−Trp, 3 mm 3‐AT, and X‐α‐gal medium. BD‐*IbZnFR^N228^* contains *IbZnFR* amino acid residues 1–228; EV, empty vector. B) BiFC assay showing that *IbCHYR1* interacts with *IbZnFR*. Scale bars, 20 µm. C) Co‐IP assay showing that *IbCHYR1* interacts with *IbZnFR*. *IbCHYR1*‐GFP and *IbZnFR*‐*HA* were individually expressed in protoplasts for 16 h and purified for the assay. Proteins were immunoprecipitated with *HA*‐trap magnetic agarose, and the immunoblots were probed with anti‐*HA* and anti‐GFP antibodies. D) The root rot ratio, and genotype and dosage of *IbZnFR* of resistant and susceptible sweet potato varieties. Data are shown as the means ± S.D. (*n* = 3). X18, Xushu18; S19, Shangshu19; XX, Xinxiang; ES11, Eshu11; ZZS2, Zhanzishu2; L9, Longshu9; P32, Pushu32; HY, Hongyao; YS5, Yanshu5; QS830, Quanshu830. The red font indicates the difference in bases within the promoter region of *IbZnFR*. cv., cultivar. E) Promoter activities of the *Pro*::*IbZnFR ^Hap1^* and *Pro*::*IbZnFR ^Hap2^* alleles. The empty vector was used as a negative control. The expression level of REN was used as an internal control. Data are presented as mean ± S.D. (*n* = 3). F) RT‐qPCR analysis of *IbZnFR* haplotypes with or without DX94 infection. The sweet potato *β‐Actin* gene was used as an internal control. Data were determined by RT‐qPCR from three biological replicates consisting of pools of five plants and are presented as the means ± S.D. (*n* = 3). G,H) Three months old field‐grown *IbZnFR*‐OE plants exhibited significantly thicker stems than in the WT plants in contrast to those in the *IbZnFR*‐Ri plants. Scale bars, 1 cm. Data are shown as the means ± S.D. (*n* = 6). I) Scanning electron microscopy image of a transversal section showing the cell wall thickness in *IbZnFR*‐OE and *IbZnFR*‐Ri transgenic plants. Scale bars, 3 µm. J,K) Fresh weight of the storage roots of *IbZnFR* transgenic and WT plants. Scale bars, 5 cm. Data are shown as the means ± S.D. (*n* = 6). In (D), (E), (H), and (K), different letters indicate statistically significant differences (one‐way ANOVA followed by a post‐hoc Tukey test; *p* < 0.05).

To elucidate the mechanisms underlying *IbCHYR1*‐mediated enhanced susceptibility to the root rot disease, we screened the Y2H library, performed immunoprecipitation assays coupled with mass spectrometry (IP–MS) using *IbCHYR1*‐OE‐E17 and WT plants, and identified 63 putative *IbCHYR1*‐interacting proteins (Table S4, Supporting Information). *IbZnFR*, which encodes a transcription factor with a C─X7─C─X5─C─X3─H‐type CCCH zinc‐finger motif (CHYFSKGFCKHGSNCRYLH) and a RRM motif and shares 98.53% homology with *IbC3H18*,^[^ ^37^ ^]^ was detected 8 times in the Y2H results and exhibited a strong interaction with *IbCHYR1* in both yeast and plant cells (**Figure** **3**A). RNA‐seq results revealed that the *IbZnFR* gene was upregulated in the two resistant varieties but downregulated in the susceptible varieties (Table S4, Supporting Information). Bimolecular fluorescence complementation (BiFC) and co‐immunoprecipitation (co‐IP) assays confirmed the interaction between *IbZnFR* and *IbCHYR1* in plant cells (Figure 3B,C).

We then measured the genotype and dosage of *IbZnFR* across all resistant and susceptible varieties using the Hi‐TOM platform. The promoter region of *IbZnFR* was mainly divided into two major haplotypes. Only the *Pro*::*IbZnFR^Hap1^* haplotype was detected in the susceptible variety (all six homologous chromosomes), whereas the *Pro*::*IbZnFR^Hap2^* haplotype was detected in the resistant variety (one to three homologous chromosomes; Figure 3D). Sequence analysis revealed that a TATA‐box binding protein‐binding motif (TTTAT) was present in the *Pro*::*IbZnFR^Hap1^* haplotype but was lost in the *Pro*::*IbZnFR^Hap2^* haplotype (Figure S5B, Supporting Information).^[^ ^38^ ^]^ Dual‐LUC reporter assays showed that the promoter activity of the *Pro*::*IbZnFR^Hap2^* haplotype was significantly higher than that of the *Pro*::*IbZnFR^Hap1^* haplotype (Figure 3E). Consistently, RT‐qPCR analysis revealed a substantial induction of the *IbZnFR^Hap2^* haplotype in resistant Xushu18, with or without DX94 infection, compared with that of the *IbZnFR^Hap1^* haplotype in susceptible Longshu9 (Figure 3F). These results suggest that the high dose haplotype *Pro*::*IbZnFR^Hap2^* is associated with root rot disease resistance.

### 2.4 *IbZnFR* Enhances Sweet Potato Resistance to Root Rot and *Fusarium* Wilt

To explore the role of *IbZnFR* in the immune response, we cloned it from the highly expressed resistant variety Xushu18 and generated overexpression and RNAi lines in Lizixiang. Notably, in vitro, *IbZnFR*‐OE and *IbZnFR*‐ Ri plants exhibited smaller leaves than in the WT plants (Figure S7, Supporting Information). Field‐grown *IbZnFR*‐OE plants exhibited significantly smaller and thicker leaves and plant stems with thicker cell walls than those in the WT and *IbZnFR*‐Ri plants (Figure 3G–I and Figure S14 (Supporting Information)). These phenotypes were the opposite of those observed for *IbCHYR1* (Figures S8 and S9A–C (Supporting Information)). Notably, *IbZnFR*‐OE plants exhibited a 9.7–26.1% increase in storage root yield (total weight of storage roots) per plant, whereas *IbZnFR*‐Ri lines displayed a 2.2–14.8% decrease compared with that in the WT plants; however, their dry matter content and storage root color remained unchanged (Figure 3J,K and Figure S15 (Supporting Information)).

**Figure 4:**
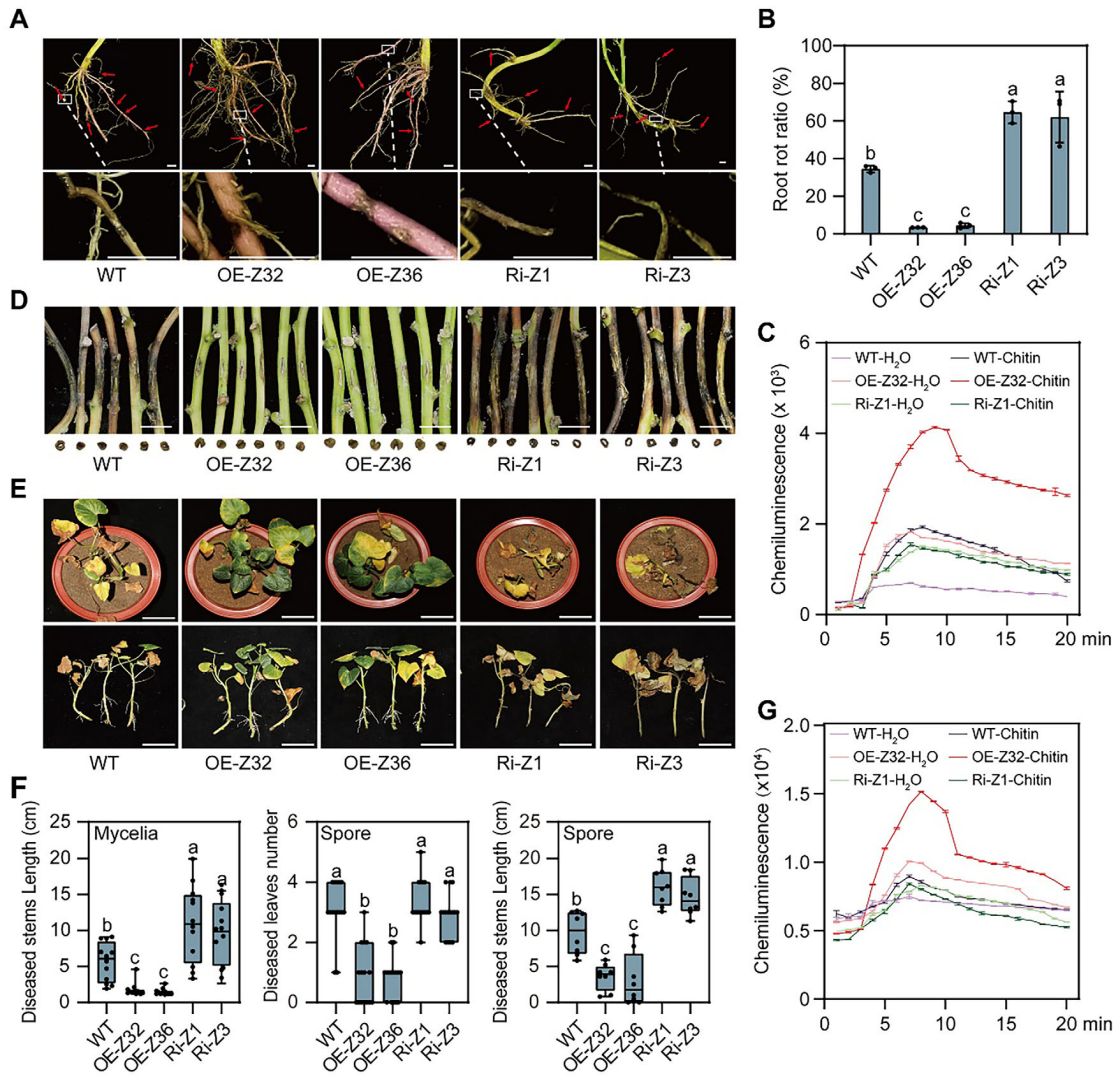
*IbZnFR* enhances sweet potato resistance to root rot and *Fusarium* wilt. A,B) *IbZnFR*‐OE lines formed less lesions than the WT, in contrast to *IbZnFR*‐Ri plants in the root rot field. Scale bars, 0.5 cm. Data are shown as the means ± S.D. (*n* = 3). C) ROS accumulation in the roots of *IbZnFR* transgenic and WT plants in the root rot field. Data are shown as the means ± S.D. (*n* = 3). D,E) Development of disease symptoms in *IbZnFR* transgenic and WT plants after *Fob* inoculation by mycelia (D, scale bars, 1 cm) or spore (E, scale bars, 10 cm). F) Statistical analysis of diseased leaves (*n* = 15) and stems (spore, *n* = 9; mycelia, *n* = 15) in *IbZnFR* transgenic plants and WT plants. Data are shown as the means ± S.D. G) ROS accumulation in the roots of *IbZnFR* transgenic and WT plants at 0 DAI and 1 DAI by the spore infection method. Data are shown as the means ± S.D. (*n* = 15). In (B), (C), (F), and (G), different letters indicate statistically significant differences (one‐way ANOVA followed by a post‐hoc Tukey test; *p* < 0.05).

Next, we assessed the root rot resistance of *IbZnFR* transgenic lines. In contrast to the *IbZnFR*‐Ri plants grown in the root rot‐infested field, the *IbZnFR*‐OE lines grown in this field displayed fewer lesions, destruction of pith and cortex structures, higher cellulose and lignin contents, and a more robust ROS burst than in the WT plants (**Figure** **4**A–C and Figures S11B, S16A,B, and S17A (Supporting Information)). Furthermore, DX94 infection indicated that the *IbZnFR*‐OE lines showed increased resistance compared to the WT, while the *IbZnFR*‐Ri plants exhibited reduced resistance (Figure S13B, Supporting Information). Moreover, after infection with *Fob*, the *IbZnFR*‐OE lines exhibited fewer diseased leaves and stem necrotic regions, less destruction of pith and cortex structures, higher cellulose and lignin contents, and a stronger ROS burst than in the WT and *IbZnFR*‐Ri plants (Figure 4D–G and Figures S11D, S16C–E, and S17B (Supporting Information)). These results suggest that *IbZnFR* enhances sweet potato yield and *Fusarium* resistance through PTI.

### 2.5 *IbCHYR1*–*IbZnFR* Complex Mediates Root Rot Response through Multiple Pathways

**Figure 5:**
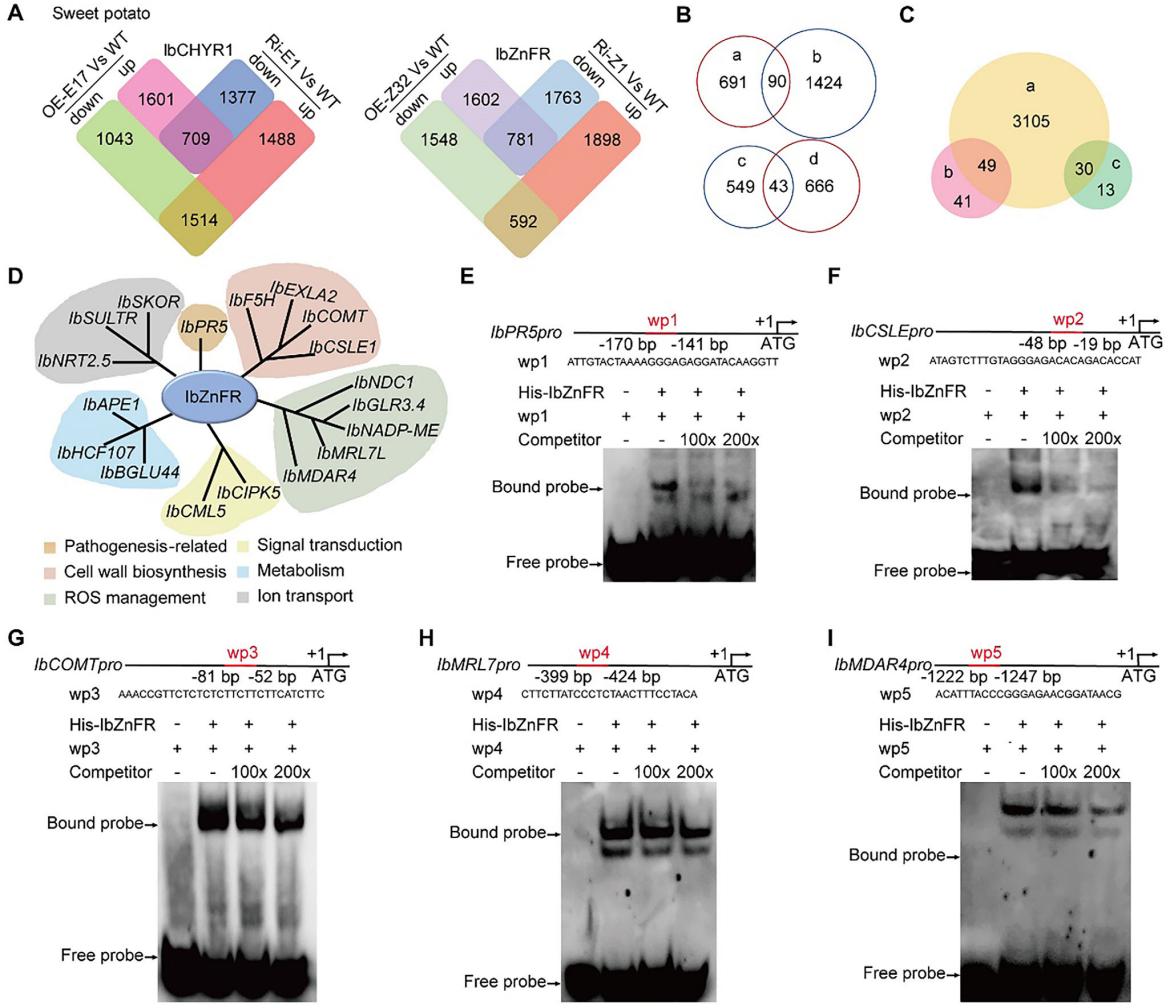
*IbCHYR1*–*IbZnFR* complex mediates root rot response through multiple pathways. A) The number of DEGs among *IbCHYR1* and *IbZnFR* transgenic plants and WT plants 10 DAP in a root rot field. All clean reads were aligned to the sweet potato genome. B) Venn diagram showing the number of DEGs among *IbCHYR1* and *IbZnFR* transgenic plants and WT plants. a) 781 DEGs were upregulated in *IbZnFR*‐OE but downregulated in *IbZnFR*‐Ri plants; b) 1514 DEGs were downregulated in *IbCHYR1‐*OE but upregulated in *IbCHYR1‐*Ri plants; c) 592 DEGs were downregulated in *IbZnFR*‐OE but upregulated in *IbZnFR*‐Ri plants; d) 709 DEGs were upregulated in *IbCHYR1‐*OE but downregulated in *IbCHYR1‐*Ri plants. C) Venn diagram showing the number and overlap of DEGs detected by RNA‐Seq and ChIP‐Seq. a) 3184 putative *IbZnFR* targets were detected by ChIP‐seq. b) 90 DEGs in (B); c) 43 DEGs in (B). D) Putative *IbZnFR* target genes affected by *IbCHYR1*. E–I) EMSA assays verified that *IbZnFR* directly binds to the promoters of pathogenesis (E), lignin/cell wall biosynthesis (F and G), and ROS management (H and I) related genes *IbPR5*, *IbCSLE1*, *IbCOMT*, *IbMRL7L*, and *IbMDAR4*. The red lines on the promoters represent the wild‐type probes.

Haplotype analysis showed that 73.02% of sweet potato accessions with the susceptible haplotype *Pro*::*IbCHYR1^Hap1^* also carried the susceptible haplotype *Pro*::*IbZnFR^Hap1^*; moreover, 51.35% of accessions with the disease‐resistant haplotype *Pro*::*IbCHYR1^Hap2^* also carried the resistant haplotype *Pro*::*IbZnFR^Hap2^*, suggesting a correlation between the distribution of these two gene haplotypes (Figure 2C and Figure S18 (Supporting Information)). To elucidate the regulatory mechanisms underlying the *IbCHYR1*–*IbZnFR*‐complex‐mediated root rot response, we performed the RNA‐seq analysis of *IbCHYR1* and *IbZnFR* transgenic plants and the WT plants at 10 DAP in the root‐rot‐infested field and aligned all clean reads to the sweet potato^[^ ^39^ ^]^ and DX94 genomes. In total 48 358 genes were found to be expressed across all *IbCHYR1* and *IbZnFR* transgenic plants and the WT plants (Figure S19, Supporting Information). Compared with that in the WT plants, 709 differentially expressed genes (DEGs) were upregulated in *IbCHYR1‐*OE but downregulated in *IbCHYR1‐*Ri plants, whereas 1514 DEGs were downregulated in *IbCHYR1‐*OE but upregulated in *IbCHYR1‐*Ri plants in the root‐rot‐infested field (**Figure** **5**A and Table S5 (Supporting Information)). Gene Ontology (GO) enrichment analysis revealed that these 2223 DEGs were enriched in processes, such as the biosynthetic process (GO:0009058), plant‐type cell wall organization (GO:0009664), the cellulose biosynthetic process (GO:0030244), and terpene synthase activity (GO:0010333) (Figure S20A, Supporting Information). Compared with that in the WT plants, 781 DEGs were upregulated in *IbZnFR*‐OE but downregulated in *IbZnFR*‐Ri plants, whereas 592 DEGs were downregulated in *IbZnFR*‐OE but upregulated in *IbZnFR*‐Ri plants in the root‐rot‐infested field (Figure 5A and Table S6 (Supporting Information)). GO enrichment analysis revealed that these 1373 DEGs were enriched in processes such as cysteine‐type peptidase activity (GO:0008234), FAD binding (GO:0071949), cellulose biosynthetic process (GO:0030244), and cell wall biogenesis (GO:0042546) (Figure S20B, Supporting Information).

Next, we explored the DEGs coregulated by the *IbCHYR1*–*IbZnFR* complex. Notably, 90 DEGs were co‐upregulated in the disease‐resistant *IbCHYR1*‐Ri and *IbZnFR*‐OE plants but co‐downregulated in the susceptible *IbCHYR1*‐OE and *IbZnFR*‐Ri plants (Figure 5B and Table S7 (Supporting Information)). Additionally, 43 DEGs were co‐upregulated in *IbCHYR1‐*OE and *IbZnFR*‐Ri plants but co‐downregulated in *IbCHYR1‐*Ri and *IbZnFR*‐OE plants (Figure 5B and Table S7 (Supporting Information)). These 133 genes may be directly regulated by the *IbCHYR1*–*IbZnFR* complex.

Using a polyclonal anti‐*IbZnFR* antibody, we identified 49 genes that were co‐upregulated in *IbCHYR1‐*Ri and *IbZnFR*‐OE plants but co‐downregulated in *IbCHYR1‐*OE and *IbZnFR*‐Ri plants by comparing the chromatin immunoprecipitation (ChIP) sequencing data for these 133 genes (Figure 5C and Table S8 (Supporting Information)). *IbZnFR* binds to the promoter regions of these genes in vivo. These genes are involved in pathogenesis, lignin and cell wall biosynthesis, ROS management, and signal transduction (Figure 5D). A subsequent electrophoretic mobility shift assay (EMSA) verified that *IbZnFR* binds directly to the promoters of *IbPR5* ^[^ ^40^ ^]^ (encoding pathogenesis‐related protein 5), *IbCSLE1* ^[^ ^41^ ^]^ (encoding cellulose synthase‐like protein E1), *IbCOMT* ^[^ ^42^ ^]^ (encoding caffeic acid 3‐*O*‐methyltransferase), *IbMRL7L* ^[^ ^43^ ^]^ (encoding thioredoxin‐like fold domain‐containing protein), and *IbMDAR4* ^[^ ^44^ ^]^ (encoding monodehydroascorbate reductase), thereby activating their expression (Figure 5E–I). These results align with the observations of stem and cell wall thickness in disease‐resistant *IbCHYR1*‐Ri and *IbZnFR*‐OE plants (Figure 3G–I and Figure S9A–C (Supporting Information)). Collectively, the *IbCHYR1*–*IbZnFR* complex mediates the root rot response through pathogenesis, lignin/cell wall biosynthesis, ROS management, and signal transduction pathways.

### 2.6 *FfRlpA2* is a Conserved Pathogenic Effector of *Fusarium* Root Rot

Pathogens secrete effectors that manipulate host immune responses and facilitate infection.^[^ ^45^ ^]^ To determine the pivotal effectors associated with *Fusarium* root rot, RNA‐seq reads of the two resistant and two susceptible sweet potato varieties were aligned with the DX94 genome (Figure 1). In the susceptible varieties, 14 effector genes were significantly upregulated compared with that in the resistant varieties (Table S9, Supporting Information). Of these, *FfRlpA2*, encoding a rare lipoprotein A, was co‐upregulated in *IbCHYR1‐*OE and *IbZnFR*‐Ri plants but co‐downregulated in *IbCHYR1‐*Ri and *IbZnFR*‐OE plants compared with that in the WT plants (Table S10, Supporting Information).

**Figure 6:**
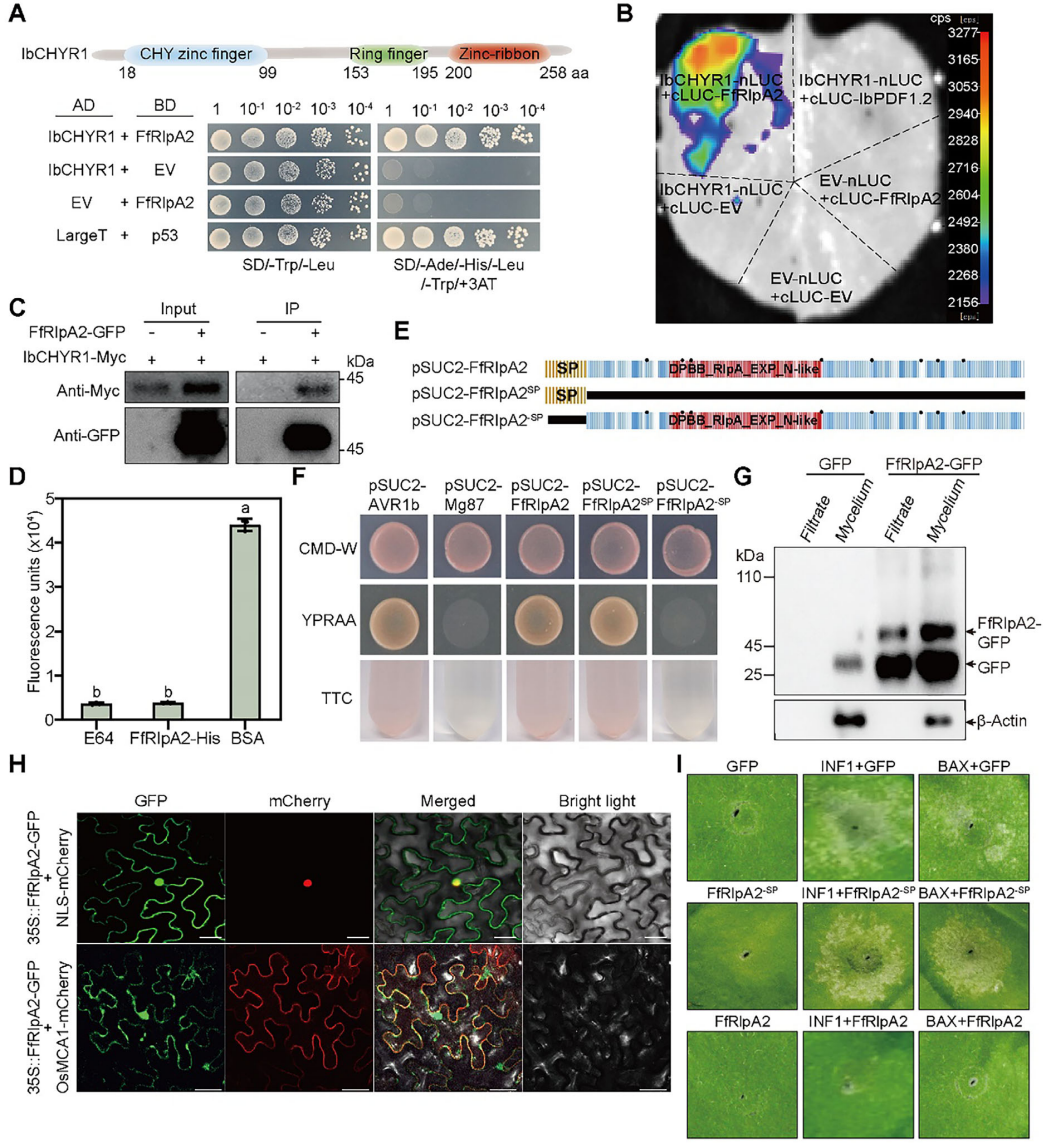
*FfRlpA2* is a conserved pathogenic effector of *Fusarium* root rot. A) *FfRIpA2* interacts with *IbCHYR1* in yeast. Yeast cells were plated onto SD/−Ade/−His/−Leu/−Trp and 3 mm 3‐AT medium. LargeT with p53 was used as positive control. EV, empty vector. B) Luciferase complementation imaging (LCI) assay showing the interaction between FfRIpA2 and *IbCHYR1* in *N. benthamiana* leaves. *IbPDF1.2* was used as a noninteracting protein for a negative control. The pseudocolor bar (right panel) shows the range of luminescence intensity in each image. C) Co‐IP assay showing that *FfRlpA2* interacts with *IbCHYR1*. *FfRIpA2*‐GFP and *IbCHYR1*‐Myc were individually expressed in protoplasts for 16 h and purified for the assay. Proteins were immunoprecipitated with GFP‐trap magnetic agarose, and the immunoblots were probed with anti‐GFP and anti‐Myc antibodies. D) Protease inhibition assay of *FfRlpA2*. The protease inhibitor E64 and BSA protein were used as positive and negative controls, respectively. Data are presented as mean ± S.D. (*n* = 3). E) Protein structure of *FfRlpA2*. Cysteine residues are marked with black points. SP, signal peptide. F) *FfRlpA2* contains a SP that drives the secretion of *suc2* mutant YTK12 cells in a yeast secretion assay. The yeast strain YTK12 was transformed with the empty vector *pSUC2* (negative control), *pSUC2*‐*FfRlpA2^−SP^* (without SP), *pSUC2*‐*FfRlpA2^SP^* (with SP), *pSUC2*‐*FfRlpA2* (with SP), or *pSUC2*‐*Avr1b* (positive control) and tested for growth on CMD‐W or YPRAA medium and for invertase activity with a colorimetric TTC assay. G) Assay for the secreting ability of *FfRlpA2*. Anti‐Actin antibody was used to confirm the protein samples of vegetative hyphae. H) *FfRlpA2* was localized to the nucleus, cell membrane, and cytoplasm in *N. benthamiana* epidermal cells (scale bars, 20 µm). The *FfRlpA2*‐GFP was coexpressed with the nuclear marker NLS‐mCherry or the plasma membrane marker OsMCA1‐mCherry. I) Assays for the suppression of BAX‐ or INF1‐induced cell death. Sweet potato leaves were infiltrated with *Agrobacterium* cells expressing GFP/BAX/INF1 or BAX/INF1‐expressing cells 18 h after GFP or *FfRlpA2*/*FfRlpA2^−SP^* infiltration. Representative leaves were photographed 7 days postinfiltration. In (D), different letters indicate statistically significant differences (one‐way ANOVA followed by a post‐hoc Tukey test; *p* < 0.05).

Y2H, firefly LUC complementation imaging (LCI), and co‐IP assays showed that *FfRlpA2* interacted with *IbCHYR1* (**Figure** **6**A–C). RT‐qPCR analysis demonstrated a marked induction of *FfRlpA2* in Longshu9 after DX94 infection, in contrast to that in Xushu18 (Figure S21, Supporting Information). Previous reports have highlighted the role of the *RsRlpA* effector as a protease inhibitor and have shown that it enhances *R. solani* virulence by suppressing the hypersensitivity response.^[^ ^22^ ^]^ Protease inhibition assays revealed that *FfRlpA2* blocked papain proteolytic activity, indicating that it functioned as a protease inhibitor (Figure 6D).

*FfRlpA2* comprises a 19‐amino acid secretion signal peptide (SP) and a conserved (DPBB)_RlpA_EXP_N‐like domain (Figure 6E and Figure S22, Supporting Information). Phylogenetic analysis revealed a high conservation of *RlpA2s* within *Fusarium* spp. (95.50% identity; Figures S22C and S23A, Supporting Information). However, their homology with other plants, bacteria, and fungi was remarkably low, suggesting a specific and general role of effectors in *Fusarium* (Figure S23, Supporting Information). Invertase secretion assays demonstrated that *pSUC2*‐*FfRlpA2^SP^* acquired the ability to grow on YPRAA medium with raffinose as the sole carbon source and reduced 2,3,5‐triphenyltetrazolium chloride (TTC) to red formazan, suggesting that SP possesses secretion capability (Figure 6F). Moreover, when expressing green‐fluorescent‐protein (GFP)‐tagged *FfRlpA2* (*FfRlpA2*‐GFP) in DX94, the GFP antibody was detectable in both the culture filtrate and the mycelium, indicating that *FfRlpA2* is a secreted protein (Figure 6G). Additionally, subcellular fluorescence and fractionation assays revealed that *FfRlpA2* was localized to the nucleus, cell membrane, and cytoplasm (Figure 6H and Figure S23C,D (Supporting Information)).

To elucidate the role of *FfRlpA2* in suppressing host immunity, we examined its impact on programmed cell death in *Nicotiana benthamiana* induced by BCL‐2‐associated protein X (BAX), a proapoptotic protein, and INF1, a PAMP from *Phytophthora infestans*.^[^ ^46^, ^47^ ^]^ The results revealed that the full‐length *FfRlpA2* effectively suppressed cell death triggered by both BAX and INF1; however, its suppressive capacity was lost without the SP domain (Figure 6I). These findings indicate that *FfRlpA2* is a pivotal and conserved pathogenic effector of *Fusarium* root rot, and that its SP domain is required for virulence.

### 2.7 *FfRlpA2* Hijacks *IbCHYR1* to Degrade *IbZnFR* and Inhibit Multiple Defense Pathways

**Figure 7:**
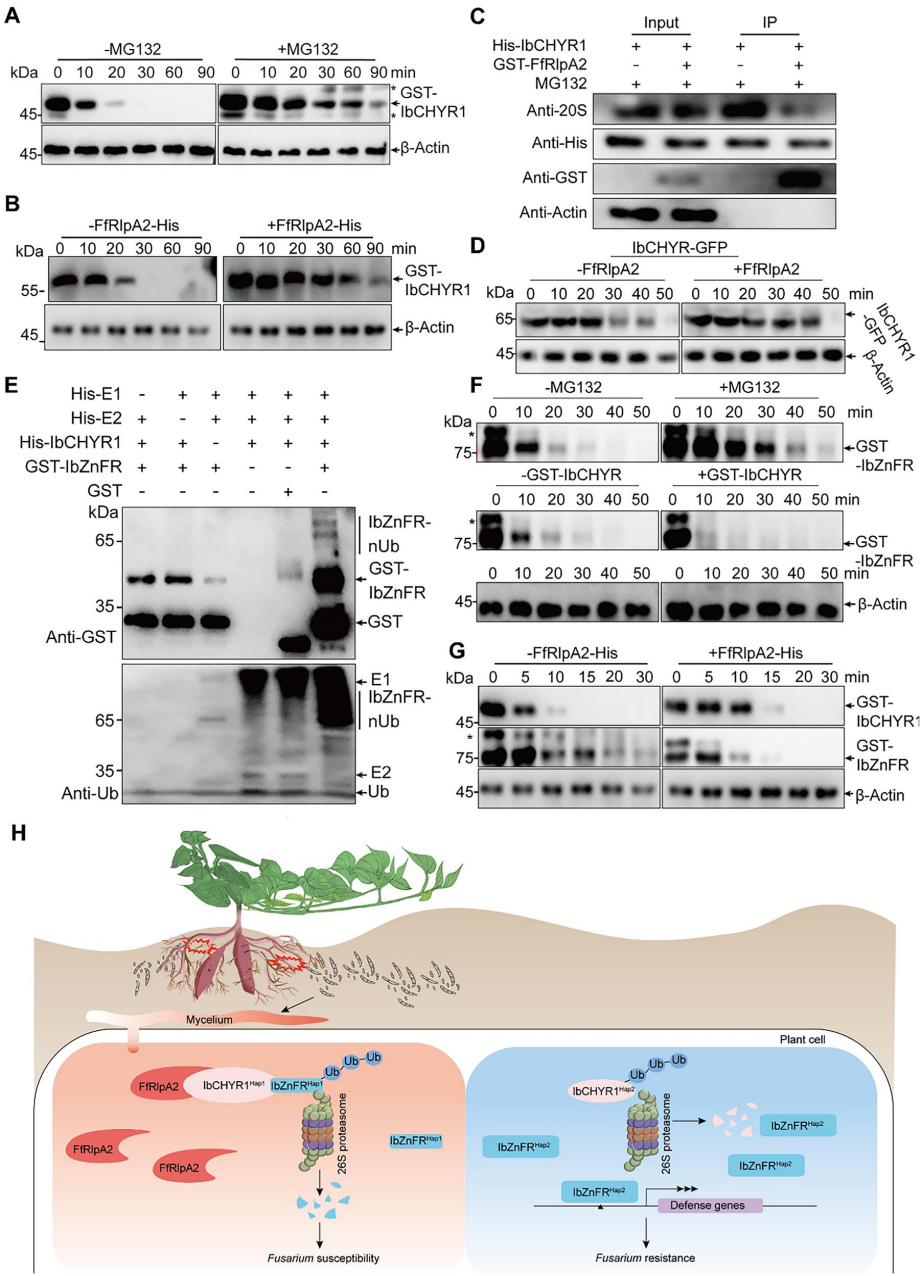
*FfRlpA2* hijacks *IbCHYR1* to degrade *IbZnFR*, leading to sweet potato root rot disease. A) Cell‐free degradation assay of *IbCHYR1*. The recombinant GST‐*IbCHYR1* protein was incubated with a crude protein extract from the WT plants. The aliquots of the mix were collected at the indicated times and probed by immunoblotting with anti‐GST antibody. B) *FfRlpA2* stabilizes *IbCHYR1*. The crude protein extract obtained from the WT plants was used for the inoculation of recombinant GST‐*IbCHYR1* and *FfRlpA2*‐His proteins. The aliquots of the mix were collected at the indicated times and probed by immunoblotting with anti‐GST antibody. C) *FfRlpA2* disrupted the interaction of *IbCHYR1* and 26S proteasome. Immunoblots of the indicated protein mixtures (GST‐*IbCHYR1* and total protein of WT) (input) or proteins copurified with *FfRlpA2*‐GFP from these protein mixtures (GST‐IP) were detected. D) *FfRlpA2* stabilizes *IbCHYR1* in *FfRlpA2‐IbCHYR1*‐OE plants. The crude protein extract obtained from the *FfRlpA2‐IbCHYR1*‐OE plants. The aliquots of the mix were collected at the indicated times and probed by immunoblotting with anti‐GFP antibody. E) In vitro ubiquitination of *IbZnFR* by *IbCHYR1*. E1, E2, *HA*‐Ubi proteins, *IbCHYR1*‐His, and GST‐*IbZnFR* were added to reactions in the presence of ATP. *IbZnFR* polyubiquitination was detected by immunoblotting with an anti‐GST antibody. Three independent experiments were performed with similar results. F) *IbZnFR* degradation was promoted by *IbCHYR1*. The crude protein extract obtained from the WT plants was used for the inoculation of recombinant *IbCHYR1*‐His and GST‐*IbZnFR* proteins. The aliquots of the mix were collected at the indicated times and probed by immunoblotting with anti‐GST antibody. G) *FfRlpA2* promotes the degradation of *IbZnFR* by *IbCHYR1*. Recombinant GST‐*IbZnFR*, GST‐*IbCHYR1*, and *FfRlpA2*‐His proteins were incubated with a crude protein extract from WT plants. The aliquots of the mix were collected at the indicated times and probed by immunoblotting with anti‐GST antibody. H) Proposed working model of the *IbCHYR1*–*IbZnFR* module responds to *FfRlpA2*‐triggered immune signaling. The *Fusarium* effector *FfRlpA2* acts as a protease inhibitor and hijacks the sweet potato E3 ubiquitin ligase *IbCHYR1* to degrade the resistance factor *IbZnFR*, thereby inhibiting multiple defense pathways such as lignin/cell wall biosynthesis and reactive oxygen species management. In (A), (C), and (F), 50 µm MG132 was added to the samples to examine protein degradation through the 26S proteasome pathway. In (A), (B), (C), (D), (F), and (G), β‐Actin was used as a loading control. Asterisk represents nonspecific band.

E3 ubiquitin ligases are degraded by the 26S proteasome.^[^ ^25^ ^]^ An E3 ubiquitin ligase activity assay showed that the recombinant *IbCHYR1* protein displayed autoubiquitination activity in the presence of E1 and E2 (Figure S24A, Supporting Information). A cell‐free degradation assay revealed that *IbCHYR1* degradation was significantly inhibited by coincubation with the proteasome inhibitor MG132, indicating dependence on the 26S proteasome (**Figure** **7**A). Notably, *FfRlpA2* effectively disrupted *IbCHYR1* degradation (Figure 7B and Figure S24B (Supporting Information)), suggesting that it targets and stabilizes the sweet potato E3 ubiquitin ligase, *IbCHYR1*. Pull‐down assays revealed that *FfRlpA2* disrupted the interaction between the histidine tagged *IbCHYR1* (*IbCHYR1*‐His) and 26S proteasome (Figure 7C). We also introduced *FfRlpA2* into the *IbCHYR1*‐OE background. Immunoblot analysis showed a significant enhancement in the accumulation of *IbCHYR1* in the *FfRlpA2*–*IbCHYR1*‐OE line compared with that in the *IbCHYR1*‐OE plants (Figure 7D).

An in vitro ubiquitination assay demonstrated that *IbCHYR1* mediates polyubiquitination of *IbZnFR* (Figure 7E). A cell‐free degradation assay showed that *IbZnFR* degradation was significantly inhibited by MG132, suggesting dependence on the 26S proteasome (Figure 7F). In addition, *IbZnFR* degradation was significantly enhanced by coincubation with *IbCHYR1* (Figure 7G and Figure S24C (Supporting Information)). These findings suggest that *IbCHYR1* interacts with *IbZnFR* to enhance its degradation.

A cell‐free degradation assay was performed to explore the relationship between *FfRlpA2* and the *IbCHYR1*–*IbZnFR* complex. In the presence of *FfRlpA2*, the degradation of *IbCHYR1* was disrupted, effectively promoting the degradation of *IbCHYR1* into *IbZnFR* (Figure 7H). Collectively, these results suggest that *FfRlpA2* hijacks *IbCHYR1* to degrade *IbZnFR*, inhibiting multiple defense pathways, and rendering sweet potato vulnerable to *Fusarium* diseases (Figure 7H).

## 3 Discussion

As a root and tuber crop, a healthy root system is crucial for the high yield and quality of sweet potato. The global spread of soil‐borne diseases poses a significant threat to agricultural production and food security, leading to up to 60% yield losses.^[^ ^48^ ^]^ Here, we emphasize the risk of root rot throughout the sweet potato life cycle, from cultivation to harvest and storage (Figure 1 and Figure S1 (Supporting Information)). Pesticides are ineffective at preventing root rot and may compromise food safety. The most efficient and sustainable way to control soil‐borne diseases is to develop disease‐resistant sweet potato varieties.

The *Fusarium* genus has high genetic variability and broad host specificity, producing potent mycotoxins that are saprotrophic and pathogenic to plants, animals, and humans.^[^ ^41^ ^]^ The dominant *Fusarium* spp. that cause sweet potato root rot vary globally, with *F. oxysporum* and *F. solani* emerging as the primary culprits.^[^ ^5^, ^6^, ^12^, ^13^, ^14^, ^15^ ^]^ We found that the effector *FfRlpA2* functions as a proteinase inhibitor, strategically hijacking the E3 ubiquitin ligase *IbCHYR1* to degrade the resistance factor *IbZnFR*, thereby inhibiting multiple defense pathways in sweet potato (Figures 2, 3, and 5, 6, 7). Although *RlpA2* proteins exhibited high conservation within the *Fusarium* genus, significant differences exist between the *RlpA2* proteins of the genus *Fusarium* and those of other taxa, including plants, bacteria, and other fungal genera (Figure S23, Supporting Information). These distinctions offer promising targets for controlling *Fusarium* infections without adversely affecting plants or beneficial soil microorganisms.

Sweet potato is a highly heterozygous, asexually propagated hexaploid, and its haplotype genome has not yet been well assembled. The accurate evaluation of haplotype dosages remains challenging. The development of simplified and cost‐effective genotyping and mutation identification methods, such as the Hi‐Tom platform,^[^ ^35^ ^]^ enables more accurate estimation of haplotypes in sweet potato. In the present study, the results of the GWAS and haplotype analysis revealed that two SNPs within the promoter region of *IbCHYR1* were associated with root rot resistance (Figure 2A,C and Table S2 (Supporting Information)). Hi‐TOM sequencing showed that high dosage of the *Pro*::*IbCHYR1^Hap2^* haplotype was associated with susceptibility to root rot (Table S2, Supporting Information). In the susceptible varieties, the enhanced promoter activity of the *Pro*::*IbCHYR1^Hap1^* haplotype led to the increased expression of *IbCHYR1*, indicating that *IbCHYR1* confers susceptibility to the root rot disease (Figure 2E and Figure S4E (Supporting Information)). Additionally, a high dosage of the *Pro::IbZnFR^Hap2^* haplotype was associated with resistance to root rot disease (Figure 3D). In the resistant varieties, the enhanced promoter activity of the *Pro*::*IbZnFR^Hap2^* haplotype led to the increased expression of *IbZnFR*, indicating that *IbZnFR* confers resistance to the root rot disease (Figure 3E,F). Our findings highlight *IbCHYR1* and *IbZnFR* as key targets for enhancing resistance in susceptible sweet potato varieties.

Plants respond quickly to a pathogen attack with a ROS burst at the invasion site, triggering immunity and preventing pathogen movement.^[^ ^49^ ^]^ A complex “ROS gene network” in plants regulates ROS generation and detoxification, thereby maintaining cellular ROS homeostasis.^[^ ^50^ ^]^ This network included proteins involved in ROS generation, such as NADPH oxidases,^[^ ^51^ ^]^ NAD(P)H dehydrogenases,^[^ ^51^ ^]^ NADP‐malic enzymes, and glutamate receptors,^[^ ^52^ ^]^ as well as proteins that facilitate ROS detoxification, including glutathione peroxidases,^[^ ^53^ ^]^ thioredoxins,^[^ ^54^ ^]^ and monodehydroascorbate reductase.^[^ ^44^ ^]^ Our data revealed that *IbZnFR* triggered an oxidative burst in sweet potato, whereas *IbCHYR1* prevented this burst (Figures 2H,L and 4C,G). The *IbCHYR1*–*IbZnFR* complex directly regulates genes associated with ROS management and participates in PTI (Figure 5D,H,I).

Host‐derived ROS strengthen cell walls or trigger intracellular signaling, stimulating phytoalexin accumulation and pathogenesis‐related protein biosynthesis.^[^ ^50^ ^]^ Plant defense responses are usually accompanied by lignification, suberization, and callose deposition, which thicken the cell wall and prevent fungal penetration.^[^ ^55^ ^]^ Our data demonstrated that the overexpression of *IbZnFR* resulted in increased cellulose and lignin contents and thicker leaves and stems, whereas *IbCHYR1*‐OE lines exhibited the opposite phenotype (Figure 3G–I and Figures S8, S9A–C, S10A,B, S14, and S16A,B, (Supporting Information)). The *IbCHYR1*–*IbZnFR* complex directly regulates the expression of the *IbCSLE1*, *IbCOMT*, and *IbF5H* genes in the immune response (Figure 5D,F,G).

Little is known about the resistance of sweet potato to fungal diseases. Overexpression of *IbBBX24* significantly enhanced *Fusarium* wilt resistance without affecting yield.^[^ ^16^ ^]^ In the present study, *IbZnFR* induced broad‐spectrum resistance to *Fusarium*, thereby improving resistance against root rot and *Fusarium* wilt while increasing yield (Figures 3J,K and 4). *IbZnFR* overexpression resulted in thicker leaves and stems, enhanced photosynthetic productivity, and more efficient transport; however, the accumulation of cell wall structural components, e.g., cellulose and lignin, promoted the expansion of storage roots, which established a superior source–sink synergistic relationship that increased yield (Figure 3G,H and Figure S14 (Supporting Information)). Hitherto, several CCCH zinc‐finger proteins have been reported to be involved in plant development and disease resistance. For instance, *AtC3H14* and *AtC3H15* regulate secondary wall thickening in *Arabidopsis*.^[^ ^56^ ^]^ Poplar *PdC3H17* and *PdC3H18* regulate secondary xylem formation.^[^ ^57^ ^]^ We previously showed that *IbC3H18* interacts with *IbPR5* in sweet potato.^[^ ^37^ ^]^ Pepper *CaC3H14* positively regulates *Ralstonia solanacearum* response.^[^ ^58^ ^]^ Rice *OsC3H12* confers resistance to bacterial blight.^[^ ^59^ ^]^ Cotton *GhZFP1* enhanced resistance to *R. solani*.^[^ ^32^ ^]^ Collectively, our findings establish *IbZnFR* as a promising candidate gene for the development of high‐yielding and *Fusarium*‐resistant elite crop varieties.

In summary, our study revealed that *FfRlpA2*, a conserved *Fusarium* effector, functions as a protease inhibitor by hijacking the E3 ubiquitin ligase *IbCHYR1* to degrade the resistance factor *IbZnFR*, thereby promoting *Fusarium* pathogenicity (Figure 7H). These findings improve our understanding of *Fusarium* pathogenicity and provide valuable insights regarding strategies and candidate genes for developing broad‐spectrum resistance to *Fusarium* in root and tuber crops without reducing yield.

## 4 Experimental Section

### Plant Materials and Growth Conditions

The *Fusarium*‐susceptible varieties Longshu9, Pushu32, and Lizixiang, *Fusarium*‐resistant varieties Xushu18 and Nongda16, and a natural population were planted at the experimental station of Beijing Guangyuan Fumin Agricultural Products Professional Cooperative, Daxing, Beijing, China. In vitro‐grown transgenic sweet potato plants were cultured on Murashige and Skoog medium at 27 ± 1 °C under a photoperiod of 13 h cool‐white, fluorescent light at 54 µmol m^−2^ s^−1^ and 11 h of dark.

### Fungal Isolation

Newly infected roots were collected, rinsed with clean water, and cut into small sections using a sterilized blade in a laminar flow cabinet. The root sections were soaked in 75% w/v alcohol for 30 s and 0.1% w/v mercury dichloride for 3 min, washed thrice using sterilized water, dried on sterilized filter paper, placed on potato dextrose agar (PDA) plates, and cultured in an incubator (25 °C, 12 h photoperiod, and 75% relative humidity). Mycelial disks (0.5 cm diameter) from fungal boundaries were obtained using a sterilized puncher and transferred to new PDA plates for pure culture.

### Phylogenetic Analyses

The fungal sequences of internal transcribed spacer, the second largest subunit of RNA polymerase II, and translation elongation factor 1‐α were amplified. MUSCLE (5.1. Linux 64) was used for polynucleotide sequence alignment, and maximum likelihood trees were constructed using MEGAX based on sequences with 1000 bootstrap repeats.

### Fungal Pathogenicity Test

The storage roots of healthy sweet potato were sterilized, and four holes were drilled evenly into each sweet potato to a depth of 2 cm using a sterilized hole punch. Mycelial disk (0.5 cm diameter) were inoculated into each hole using a sterilized puncher. All media were moistened with sterilized water and cultured for 7 to 10 days at 28 °C. The negative control was inoculated with the same volume of the PDA medium.

### Transcriptome Analyses

RNA was extracted from the roots of two resistant varieties (Xushu18 and Nongda16), two susceptible varieties (Longshu9 and Pushu32), *IbCHYR1* and *IbZnFR* transgenic plants, and WT plants 10 DAP in the root rot field. Three independent biological replicates were analyzed. Libraries were generated and sequenced using the MGI2000 system as paired‐end 150 bp reads according to the manufacturer's instructions. Clean reads were mapped to the genomes of wild sweet potato relative to those of *Ipomoea triloba* NCNSP0323^[^ ^39^ ^]^ or DX94. A false discovery rate of <0.05, as determined by DESeq2, indicated differential expression.^[^ ^60^ ^]^

### Subcellular Localization

The coding regions of *IbCHYR1* and *FfRlpA2*, without the stop codons, were cloned into the pCAMBIA1300‐GFP binary vector. The plasmids were transformed into *Agrobacterium tumefaciens* strain GV3101 (pSoup‐p19). Fluorescent signals were detected using a confocal laser‐scanning microscope (LSM900, Zeiss Co., Ltd., Oberkochen, Germany) with excitation at 488 and 561 nm, NLS‐mCherry and OsMCA1‐mCherry were used as a nuclear marker and a membrane marker, respectively.^[^ ^16^, ^61^ ^]^ The experiment was repeated 3 times with similar results. The membrane protein fractions were extracted using a membrane protein and cytoplasmic protein extraction kit according to the manufacturer's protocol (Beyotime Biotechnology, P0033). Total protein from the membrane or cytoplasm was separated by sodium dodecyl sulfate–polyacrylamide gel electrophoresis and transferred to a PVDF membrane; H^+^‐ATPase was used as a membrane‐localized positive control.^[^ ^62^ ^]^ The samples were immunoblotted with anti‐GFP (EASYBIO, BE2070, 1:5000 dilution), anti‐H^+^‐ATPase (Agrisera, AS07260‐ALP, 1:10000 dilution).

### Expression Analysis

Total RNA was extracted from roots after inoculation with DX94 at a spore density of 1.5 × 10^7^ mL^−1^. Three biological replicates, each containing five plants, were used. The transcript levels were measured using RT‐qPCR, with the sweet potato *`*β‐actin gene (GenBank AY905538) and the *F. foetens* actin gene (*FfActin*, GenBank XP 031048258.1) as internal controls. Gene expression was quantified using the comparative C_T_ method.^[^ ^63^ ^]^

### Root Rot Resistance Assays

Stem cuttings (20 cm, without roots) of the natural population, transgenic, and WT plants were planted in a field with root rot soil at Daxing, China Agricultural University, Beijing, China. An adjacent field without root rot was used as the negative control. Three biological replicates consisting of pools of three plants were used. For DX94 inoculation, 300 g of wheat grain and 200 mL of water were mixed and sterilized using steam in autoclavable bags. Half a plate of DX94 mycelia was transferred into each sterilized grain bag and cultured for 7 days. Ten wheat grains containing DX94 were placed next to each seedling for inoculation. After incubation for 7 days at 37 °C, disease symptoms were evaluated.

### GWAS and Haplotype‐Based Association Analyses

A VCF file containing 4 599 509 SNPs for 314 germplasm lines of a natural sweet potato population was downloaded from https://zenodo.org/records/7184909. These SNPs were filtered based on Hardy–Weinberg equilibrium using PLINK^[^ ^64^ ^]^ (v1.90b6.21; *P* < 1.0 × 10^−6^). A GWAS was performed with 1 716 948 filtered SNPs using a mixed linear model by GAPIT.^[^ ^65^ ^]^ LD blocks were defined as groups of SNPs with a correlation coefficient (*R* ^2^) > 0.2 linked to significant loci using PLINK, with the block size determined by the physical distance between them. A pairwise LD diagram was drawn using LDBlockShow v1.40.^[^ ^66^ ^]^ Haplotype blocks between each germplasm were extracted and filtered using R script. Violin maps were drawn based on the genotype and the corresponding phenotypic data using R script. Root rot phenotypes among different haplotypes were compared using Student's *t*‐test.

### Hi‐TOM Sequencing

Genomic DNA was extracted from sweet potato leaves using the cetyltrimethylammonium bromide method. The first‐round PCR was performed to amplify the targeted genomic region using a pair of site‐specific primers with common bridging sequences added at the 5′ end (Table S1, Supporting Information). The 50 µL reaction mix contained 200 ng genomic DNA, 0.4 µm specific primers, and 25 µL 2 × Exp Taq Master Mix Ver.2 (AGBIO, AG11409). The following PCR conditions were used: incubation for 1 min at 94 °C (1×), 30 s at 98 °C, 30 s at the annealing temperature, and 30 s at 72 °C (33×), followed by incubation at 72 °C for 2 min. The PCR products were sent to the Hi‐TOM platform for sequencing. Finally, 2000 clean reads of PCR products with a 1% filter threshold from secondary amplification were sequenced on an Illumina sequencer and further decoded using the Hi‐TOM platform (http://www.hi‐tom.net/hi‐tom/).

### Dual‐LUC Assay

The promoter sequences were individually inserted upstream of the firefly LUC reporter gene into the pGreenII 0800‐LUC vector to generate reporters. An empty pGreenII 0800‐LUC vector was used as the negative control. Rice protoplasts were isolated and used for dual‐LUC assays as described previously.^[^ ^67^ ^]^ LUC and *Renilla* LUC (REN) activity levels were measured using a Dual‐LUC Reporter Assay System (Promega). LUC activity was normalized to the REN activity. Three biological replicates were used for each analysis.

### Production of Transgenic Plants

The pCAMBIA1300‐*IbCHYR1*‐GFP and pCAMBIA3301‐*IbZnFR* constructs and the empty pCAMBIA1300‐GFP vector were transfected into the *A. tumefaciens* strain EHA105. Transformation and plant regeneration were performed using embryogenic suspension cultures of sweet potato of the Lizixiang variety as previously described.^[^ ^16^ ^]^ The recombinant pCAMBIA 1307 vector containing the coding region of the *FfRlpA2* gene fused with the coding region of the hemagglutitin (*HA*) gene (pCAMBIA1307‐*FfRlpA2*‐*HA*) and the empty pCAMBIA1307‐*HA* vector were transfected into *A. tumefaciens* strain EHA105 and transformed into *IbCHYR1*‐OE plants.

### *Fusarium* Wilt Resistance Assay

Fungal pathogen *Fob* cultures were incubated in the dark at 28 °C on PDA plates for 1 week before use. For spore infection, transgenic and WT stem cuttings (12 cm, without roots) were dipped in a 1.5 × 10^7^ mL^−1^ spore solution for 30 min and cultivated in sterilized sand with sterilized Hoagland solution. For mycelial infection, *Fob* mycelial disks (1 cm in diameter) were obtained from the PDA plates. A 1 cm long wound was made in the stem using a sterile blade, and a mycelial disk was placed over the wound using sterile cotton. Sterile PDA disks served as controls. Relative humidity was maintained at 99%. The number of diseased leaves and length of the necrotic regions on the wounded stems were recorded.

### Measurement of Resistance Indices

Cellulose and lignin contents were measured using a Cellulose Content Assay Kit and a lignin assay kit (Comin Biotechnology Co., Ltd., Suzhou, China) according to the manufacturer's protocol. Oxidative burst detection in sweet potato seedlings was performed as described previously.^[^ ^68^ ^]^ Root and stem cuttings (4 and 1 mm long, respectively) from 5 weeks old sweet potato plants were floated in H_2_O overnight. After the root and stem cuttings were dried on a paper towel, they were submerged in a 96‐well plate (one disk per tube) containing 100 µL of luminescence detection buffer (0.02 mm luminol, 10 µg mL^−1^ chitin, and 20 µg mL^−1^ horseradish peroxidase). The luminescence was measured at 535 nm (excitation at 490 nm) for 20 min using a TECAN Infinite F200 microplate reader (TECAN). Stem and root samples were collected for histological examination, as described by Chai et al.^[^ ^56^ ^]^ Sweet potato stem samples were plated with gold and observed under a scanning electron microscope (SU8010; Hitachi).

### Measurement of Dry Matter and Color Index Values of Storage Roots

Except for the two plants at each end of each plot, five plants were randomly selected to measure the fresh yield. A 100 g mixed fresh slices sample was weighed and then dried at 105 °C for 30 min, at 60 °C for 5 h, and finally at 80 °C for 48 h.^[^ ^69^ ^]^ The color of the surface of the fresh‐cut storage root was measured using a Minolta Chromameter (CR‐20, Minolta, Tokyo, Japan). The color variables, L* (whiteness/darkness), a* (redness/greenness), and b* (yellowness/blueness), were measured at three locations on each sample. The results were expressed as the mean value from three replications measured from three samples of fresh‐cut storage root. The color index values were calculated using the following equations.^[^ ^70^ ^]^

### Protein Interaction Assays

The Y2H assay was performed as described in the Yeast Protocols Handbook (Clontech). *FfRlpA2* and *IbZnFR* ^N228^ coding sequences were cloned into the pGBKT7 vector (Invitrogen, Waltham, MA, USA), and full‐length *IbCHYR1* was cloned into pGADT7. These constructs were cotransformed into the yeast strain Y2H Gold using the lithium acetate method. Transformants were plated on synthetic defined medium (SD) lacking tryptophan and leucine (SD/−Trp/−Leu), and positive clones were selected on SD/−Trp /−Leu/−His/−Ade/+3AT/+X‐a‐gal.

The IP–MS assay was performed as described previously.^[^ ^71^ ^]^ Briefly, total protein was extracted from 4 weeks old OE and WT plants (≈5 g leaf fresh weight) using lysis buffer and precipitated with Dynabeads protein G conjugated with anti‐GFP antibodies (1:500 dilution, M20004; Absmart). The complexes were then washed and subjected to sodium dodecyl sulfate–polyacrylamide gel electrophoresis. Proteins were digested in a gel using MS‐grade Trypsin Gold (Promega) following the manufacturer's instructions. Before liquid chromatography–tandem mass spectrometry analysis, the IP eluate was further processed.

For the BiFC assays, the coding sequence of *IbZnFR* was cloned into pSPYNE‐35S and fused to the N‐terminus of yellow fluorescent protein (YFP), and the coding sequence of *IbCHYR1* was cloned into pSPYCE‐35S and fused to the C‐terminus of YFP.^[^ ^72^ ^]^ These vectors were transformed into *A. tumefaciens* strain GV3101 and coinfiltrated into *N. benthamiana* leaves. After 48 h, yellow fluorescence was observed using a confocal laser‐scanning microscope (LSM880, Zeiss) with an argon laser (514 nm excitation).

For the LCI assays, the coding sequences of *FfRlpA2* and *IbPDF1.2* were separately cloned into the C‐terminus of the LUC reporter gene, and the coding sequence of *IbCHYR1* was cloned into the N‐terminus of the LUC reporter gene.^[^ ^73^ ^]^ These constructs were coinfiltrated into *N. benthamiana* and the infiltrated leaves were analyzed for LUC activity 48 h postinfiltration using chemiluminescence imaging (NightShade LB985; Berthold).

For the co‐IP assays, *FfRlpA2*‐GFP, *IbCHYR1*‐MYC, *IbCHYR1*‐GFP, and *IbZnFR*‐*HA* were coexpressed in protoplasts for 16 h before being harvested for extraction with IP buffer.^[^ ^16^ ^]^ Proteins were incubated with GFP agarose (GTA‐20; Chromotek) at 4 °C for 2 h and washed 5 times with IP buffer. *FfRlpA2*‐GFP, *IbCHYR1*‐MYC, *IbCHYR1*‐GFP, and *IbZnFR*‐*HA* were detected using anti‐GFP (1:5000 dilution, BE2002; EASYBIO), anti‐MYC (1:5000 dilution, BE2011; EASYBIO), and anti‐*HA* (1:5000 dilution, BE2008; EASYBIO) antibodies.

### ChIP Assay

The ChIP assay was performed as described by Zhang et al.^[^ ^16^ ^]^ Briefly, roots (≈2 g) of OE‐32‐ and empty vector‐transformed plants were cross‐linked in 1% w/v formaldehyde under vacuum. Cross‐linking was stopped by adding 0.125 m glycine. The samples were ground to a powder in liquid nitrogen and subjected to nuclear isolation. Anti‐*IbZnFR* (1:1500 dilution) antibodies were used to immunoprecipitate the protein–DNA complexes, and the precipitated DNA was recovered. An equal amount of the chromatin sample without antibody precipitation was used as the input control. ChIP DNA was analyzed by real‐time PCR (qPCR) and ChIP values were normalized against the values of the respective inputs.

### Electrophoretic Mobility Shift Assay

The pET‐28a‐*IbZnFR* construct was introduced into *Escherichia coli* Transetta (DE3) cells to produce recombinant His‐*IbZnFR*. The recombinant protein was purified as previously described.^[^ ^16^ ^]^ Probes labeled with biotin at the 5′ ends were used as binding probes. EMSAs were performed using a LightShift Chemiluminescent EMSA Kit (Thermo Fisher Scientific, Waltham, MA, USA) following the manufacturer's instructions.

### Protease Inhibition Assay

The protease inhibition assay was performed as described previously by measuring the enzymatic activity of Z─Phe─Arg─Nmec.^[^ ^22^ ^]^ 10 µm of purified *FfRlpA2* protein was mixed with 5 µm Z─Phe─Arg─NMec (Merck, Kenilworth, NJ, USA) and 5 µm papain (Sigma‐Aldrich, St. Louis, MO, USA) in 850 µL of total reaction buffer (0.1 m buffer KH_2_PO_4_/Na_2_HPO_4_, pH 6.8; 4 mm cysteine; 1 mm Na_2_EDTA; 200 mm NaCl; 0.05% Brij 35). 10 µm of cysteine protease inhibitor E‐64 (Sigma‐Aldrich, St. Louis, MO, USA) or BSA protein were used as a positive and negative control, respectively.

### Invertase Secretion Assay

*FfRlpA2* was analyzed using SignalP 6.0.^[^ ^74^ ^]^ The predicted SP of *FfRlpA2* was cloned into a *pSUC2* vector carrying the yeast *SUC2* gene without the SP sequence.^[^ ^75^ ^]^ The SP‐*SUC2* construct was transformed into the yeast *SUC2* mutant YTK12 and tested for growth on CMD‐W and YPRAA media.^[^ ^76^, ^77^ ^]^ YTK12 transformants carrying an empty *pSUC* vector or *pSUC2*‐*Avr1b* were used as negative and positive controls, respectively.^[^ ^78^ ^]^ Invertase enzymatic activity was detected by the reduction of TTC to the insoluble, red‐colored 1,3,5‐triphenylformazan.^[^ ^79^ ^]^

### Assay for the Secreting Ability

pCAMBIA1300‐*FfRlpA2*‐GFP and pCAMBIA1300‐GFP were separately transfected into *A. tumefaciens* strain AGL1 and transformed into DX94. Transformants were screened on PDA plates containing hygromycin B (250 µg mL^−1^, Roche). The proteins from vegetative hyphae or culture medium of the transformants were incubated with GFP agarose (Chromotek, GTA‐20) at 4 °C for 2 h. Total proteins extracted from the mycelia and the culture filtrates were immunoblotted using an anti‐GFP antibody (EASYBIO, BE2070, 1:5000 dilution).

### Assays for the Suppression of BAX‐ or INF1‐Induced Cell Death

pCAMBIA1300‐*FfRlpA2/FfRlpA2^−SP^* was separately transformed into *A. tumefaciens* strain GV3101 to express BAX and INF1 constructs.^[^ ^80^, ^81^, ^82^ ^]^ At 18 h after the initial infiltration of tobacco leaves by the *A. tumefaciens* transformant carrying the 35S‐*FfRlpA2/FfRlpA2^−SP^* construct, the same sites were infiltrated with cells carrying the GFP, BAX, or INF1 constructs.^[^ ^82^ ^]^ Plant cell death symptoms were examined 7 days after infiltration. Each infiltration experiment was repeated at least 3 times with a minimum of three leaves.

### In Vitro Ubiquitination Assay

The recombinant *IbCHYR1*‐His, glutathione S‐transferase (GST)‐tagged *IbCHYR1* (*IbCHYR1*‐GST), *FfRlpA2*‐His, E1 (His‐AtUBA1), E2 (His‐UbcH5b), GST‐*IbZnFR*, and *IbZnFR*‐His were expressed in *E. coli* BL21 (DE3) and purified using Ni Sepharose and Glutathione Sepharose, respectively. The proteins were eluted with 150 mm imidazole or 10 mm GSH. E1, E2, *HA*‐Ub, and His‐*IbCHYR1* were incubated in ubiquitination reaction buffer at 30 °C with oscillations for 2 h.^[^ ^83^ ^]^ For *IbZnFR* polyubiquitination, GST‐*IbZnFR* was added. After the reaction, samples were added to 5× protein loading buffer and boiled at 95 °C for 5 min. The samples were immunoblotted with anti‐Ub (EASYBIO, BE4002, 1:5000 dilution), anti‐His (EASYBIO, BE2017, 1:5000 dilution), and anti‐GST (EASYBIO, BE2014, 1:5000 dilution) antibodies.

### Pull‐Down Assay

*IbCHYR1*‐His was incubated with total protein extracts from sweet potato in the presence or absence of *FfRlpA2*‐GST for 60 min before the addition of anti‐His beads. Following the elution of the proteins bound to the anti‐His beads, the 26S proteasome was detected using an anti‐20S proteasome antibody (Abcam, ab22673, 1:10 000 dilution).^[^ ^84^ ^]^

### Detecting Protein Stability

Total proteins were extracted from 30 days old WT plants using native buffer (50 mm Tris‐HCl pH 8.0, 10 mm EDTA pH 8.0, 0.5 m Sucrose, 1 mm MgCl_2_, and 5 mm DTT). GST‐*IbCHYR1* or GST‐*IbZnFR* was incubated with reaction buffer (equal amounts of total proteins in the presence of 1 mm ATP) at 30 °C for the specified time.^[^ ^83^ ^]^ Samples were treated with 50 µm MG132 to examine protein degradation. GST‐*IbCHYR1* or GST‐*IbZnFR* was detected by immunoblotting using an anti‐GST antibody (EASYBIO, BE2014, 1:5000 dilution), with β‐Actin (Lablead, BP0101, 1:5000 dilution) as the internal control.

### In Vivo and Cell‐Free Degradation Assay

Total proteins were extracted from 30 days old WT plants using a native buffer. Equal amounts of GST‐*IbCHYR1* protein were incubated with reaction buffer at 30 °C in the presence or absence equal amounts of *FfRlpA2*‐His protein.^[^ ^83^ ^]^ Additionally, equal amounts of GST‐*IbZnFR* proteins were incubated with reaction buffer at 30 °C in the presence or absence equal amounts of *IbCHYR1*‐His protein. Furthermore, equal amounts of GST‐*IbCHYR1* and GST‐*IbZnFR* proteins were incubated with reaction buffer at 30 °C in the presence or absence equal amounts of *FfRlpA2*‐His protein. *IbZnFR*‐His proteins were incubated with reaction buffer at 30 °C in the presence or absence equal amounts of *FfRlpA2*‐His protein, which was used as a native control. An aliquot of the mix was collected at the indicated time points, and GST‐*IbCHYR1*, GST‐*IbZnFR*, and *IbZnFR*‐His were detected by immunoblotting using an anti‐GST antibody (EASYBIO, BE2014, 1:5000 dilution) and anti‐His (EASYBIO, BE2017, 1:5000 dilution), with β‐Actin (Lablead, BP0101, 1:5000 dilution) as the internal control.

### Statistical Analyses

All data were analyzed using the one‐way analysis of variance (ANOVA) and Student's *t*‐test using SPSS 26.0. Data were shown as means ± standard deviation.

## Conflict of Interest

The authors declare no conflict of interest.

## Author Contributions

H.Z., Z.D., X.C.Z., and M.S. contributed equally to this work. H.Z. and S.H. conceived and designed the research; H.Z., Z.D., X.C.Z., M.S., X.G., and X.L.Z. performed the experiments; H.Z., Z.D., X.C.Z., M.S., X.G., R.M., L.Z., and N.Z. analyzed the data; H.Z. wrote the paper; Q.L., H.Z., S.G., Q.C., Q.L., and S.H. revised the paper; and all authors read and approved the final version of the paper.

## Supporting information



Supporting Information

Supplemental Table 1

Supplemental Table 2

Supplemental Table 3

Supplemental Table 4

Supplemental Table 5

Supplemental Table 6

Supplemental Table 7

Supplemental Table 8

Supplemental Table 9

Supplemental Table 10

## Data Availability

All data needed to evaluate the conclusions in the paper are present in the paper and/or the Supporting Information. The RNA‐seq data of *IbCHYR1* and *IbZnFR* transgenic plants, Hi‐Tom sequencing data of *IbCHYR1* and *IbZnFR* in 13 resistant and susceptible sweet potato varieties, and the ChIP‐seq data of *IbZnFR* can be accessed in the NCBI database under accession number PRJNA1151202.
