# Correction to “[Natural Allelic Variations in *IbCHYR1*–*IbZnFR* Complex Regulate *Fusarium* Root Rot Resistance in Sweet Potato]”

**DOI:** 10.1002/advs.202520146

**Published:** 2025-10-30

H. Zhang, Z. Dai, X. Zhang, et al. “Natural Allelic Variations in *IbCHYR1*–*IbZnFR* Complex Regulate *Fusarium* Root Rot Resistance in Sweet Potato.” adv. Sci. 12, no. 33 (2025): 12, e15202.

https://doi.org/10.1002/advs.202415202

The β‐Actin image in Figure 7G has been replaced due to its duplication with that in Figure 7B in the previous version. The correct version of Figure 7 is provided below.


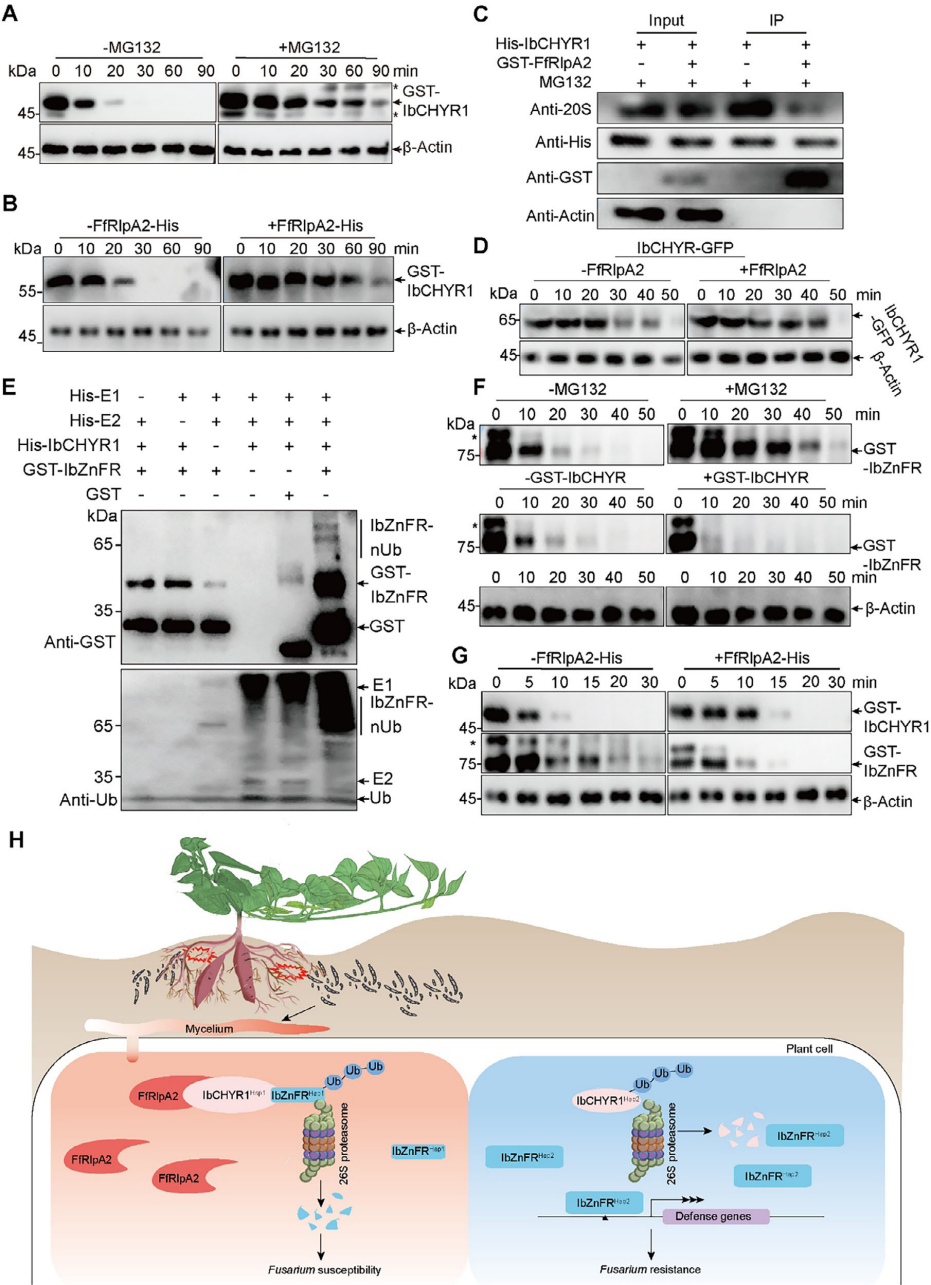


We apologize for this error.

