# Supplementary material for: Natural Allelic Variations in IbCHYR1–IbZnFR Complex Regulate Fusarium Root Rot Resistance in Sweet Potato
Source: Adv Sci (Weinh). 2025 Jun 26;12(33):e15202. doi: 10.1002/advs.202415202 (PMC12412572; doi:10.1002/advs.202415202)
Supplement: Supplementary file 1 — Supporting Information [file ADVS-12-e15202-s003.docx]

Supporting Information

**Natural allelic variations in IbCHYR1-IbZnFR complex regulate *Fusarium* root rot resistance in sweet potato**

*Huan Zhang*^†*^, *Zhuoru* *Dai*^†^, *Xiaochen* *Zhang*^†^, *Meiqi* *Shang*^†^, *Xiaoru Gao*, *Ruiqi Ma*, *Limeng Zhao*, *Xiaoli Zhang*, *Qingchang Liu*, *Hong Zhai*, *Shaopei Gao*, *Ning Zhao*, *Qinghe Cao*^*^, *Qiang Li*^*^, *Shaozhen He*^*^


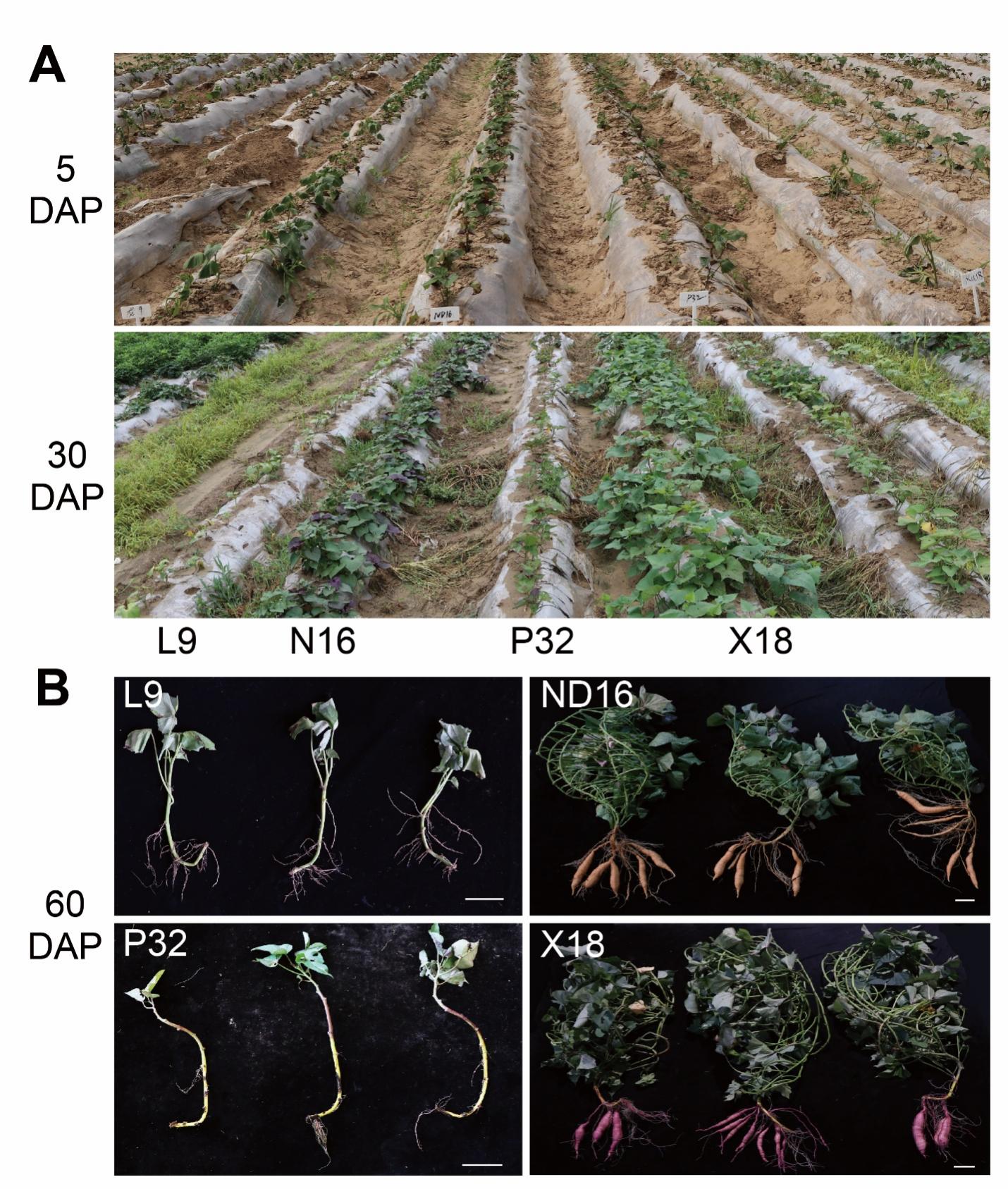


**Figure S1.** **The entire growth period of sweet potato is threatened by root rot disease.** Two root rot resistant (X18 and N16) and two susceptible (L9 and P32) varieties planted in a severe root rot-infested field in Daxing, Beijing are shown 5, 30, and 60 days after planting (DAP). X18, Xushu18; N16, Nongda16; L9, Longshu9; P32, Pushu32. Scale bars represent 5 cm.

**
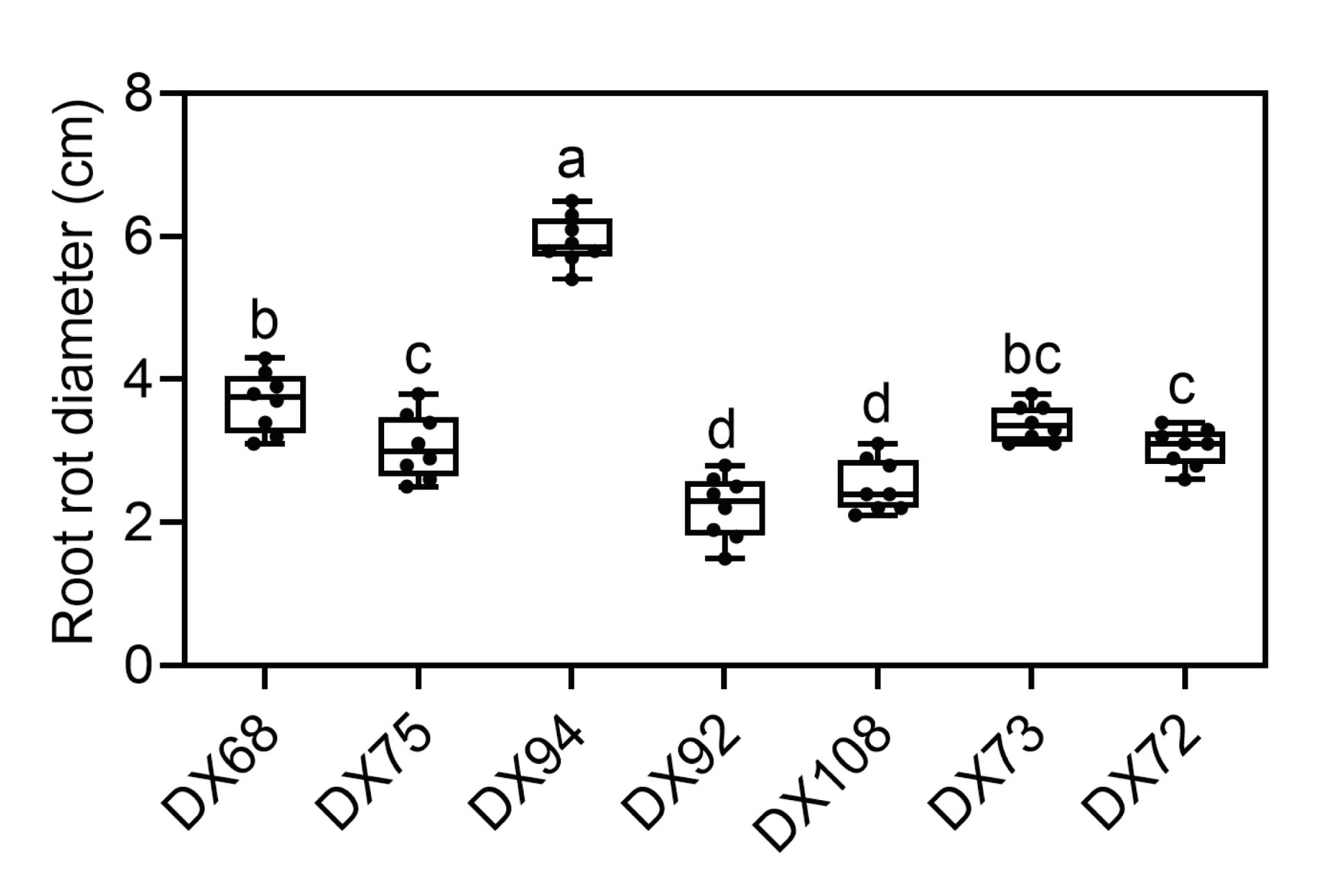
**

**Figure S2.** **Pathogenicity tests for seven fungal strains from root rot plants in Daxing, Beijing.** Data are shown as the means ± SD (*n* = 10). Different letters indicate statistically significant differences (one-way ANOVA followed by a *post-hoc* Tukey test; *P* < 0.05).

**
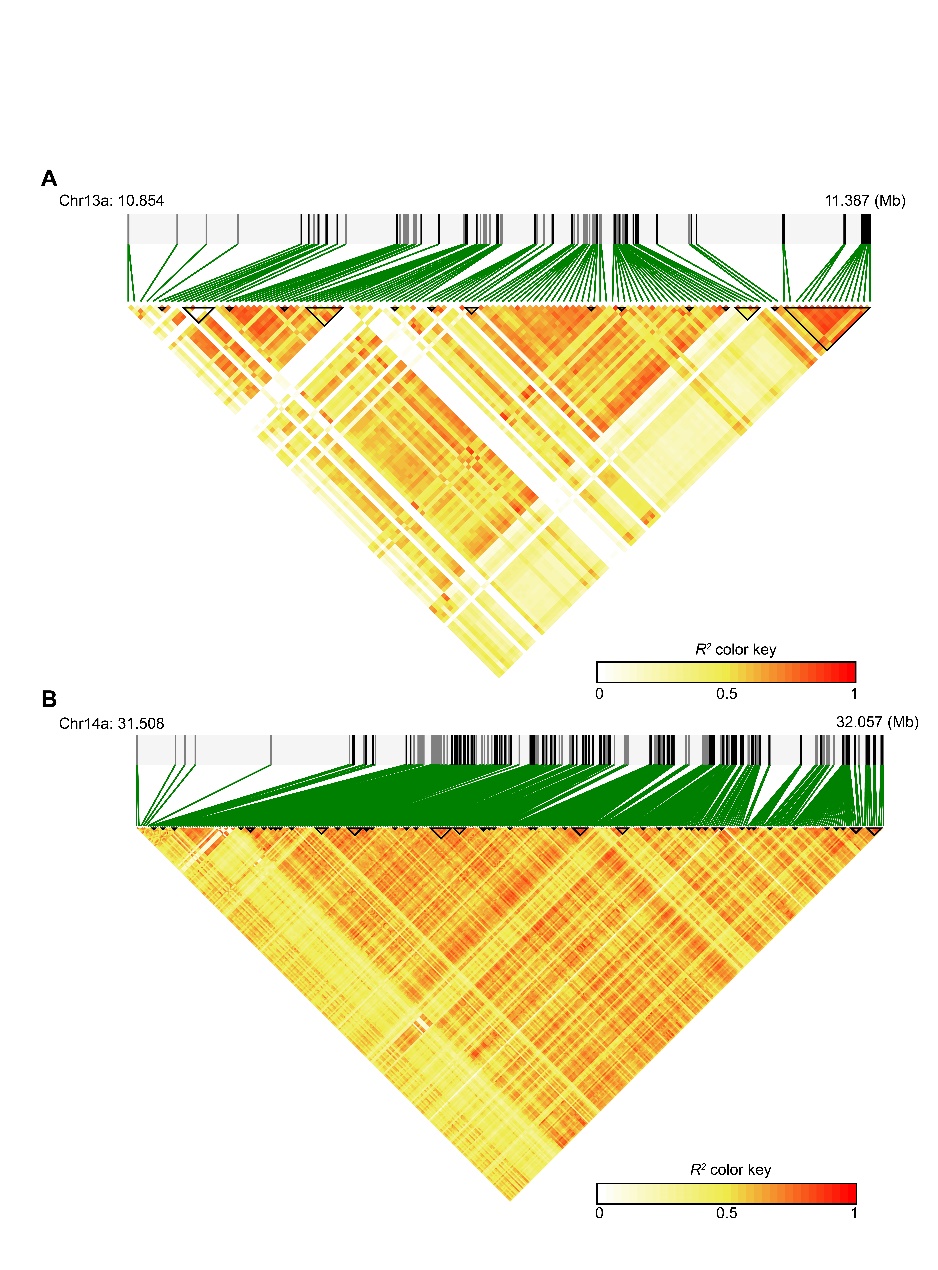
**

**Figure S3. Linkage disequilibrium blocks of Iba_chr13a_11097067 (A) and Iba_chr14a_31763570 (B), along with the corresponding LD *R^2^* partern.**


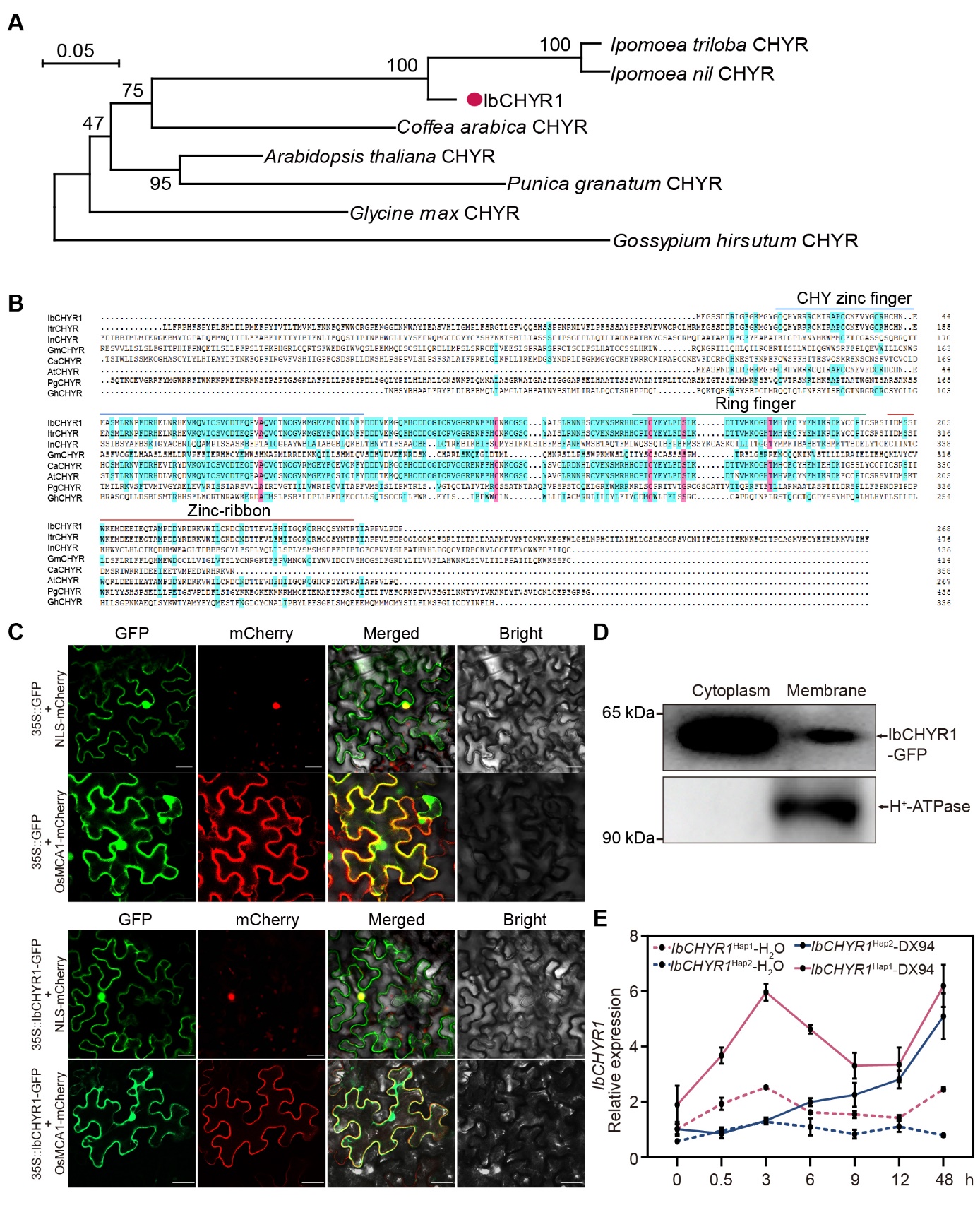


**Figure S4. Phylogenetic analysis, subcellular localization, and expression of IbCHYR1.** A) Phylogenetic analysis of CHYR proteins performed using the neighbor-joining method in MEGA6.0 with 1,000 bootstrap iterations. The numbers at the nodes of the tree indicate the bootstrap values of 1,000 replicates. B) Multiple protein sequence alignment of CHYR proteins, with conserved amino acids shaded in different colors. The CHY-zinc-finger, RING-finger, and zinc-ribbon domains are outlined. C) IbCHYR1 was localized to the nucleus, cell membrane, and cytoplasm in *Nicotiana benthamiana* epidermal cells. The IbCHYR1-GFP was co-expressed with the nuclear marker NLS-mCherry or the plasma membrane marker OsMCA1-mCherry. The pCAMBIA1300-GFP vector was used as a native control. Scale bars, 20 μm. D) Subcellular fractionation assays of IbCHYR1. H⁺-ATPase was used as a membrane-localized positive control. E) RT-qPCR analysis of *IbCHYR1* haplotypes with or without DX94 infection. The sweet potato *β-Actin* gene was used as an internal control. Data were determined from three biological replicates consisting of the pools of five plants and are presented as the means ± standard deviation (*n* = 3).


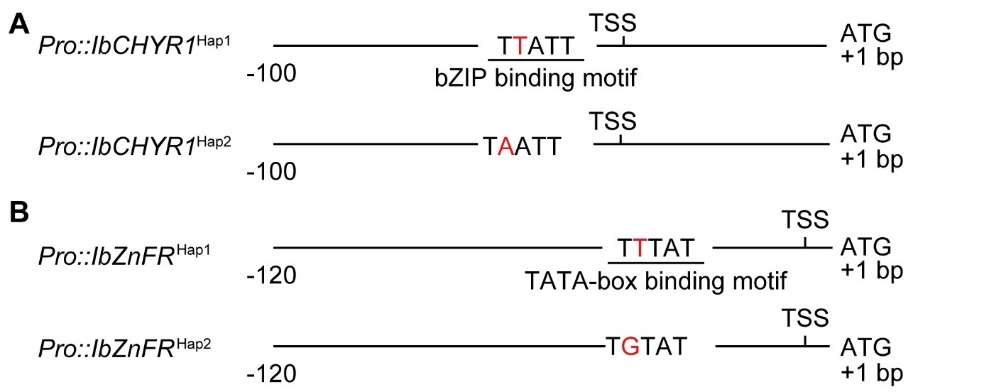


**Figure S5.** **Differences in *cis*-elements among the promoters of different haplotypes of *IbCHYR1* (A) and *IbZnFR* (B)*.*** TSS: transcription start site.


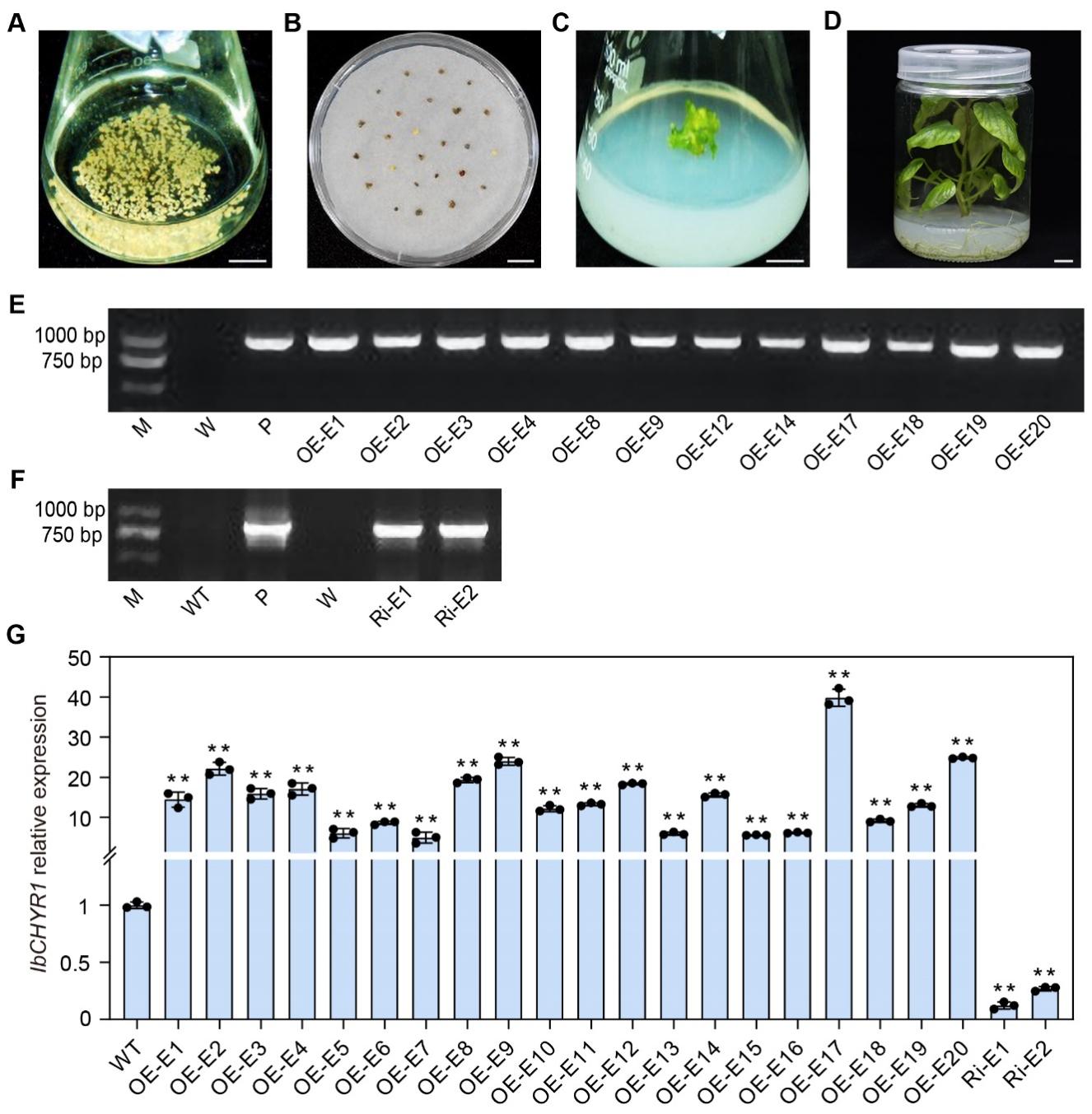


**Figure S6. Production of *IbCHYR1* transgenic sweet potato plants.** A) Proliferation of embryogenic suspension cultures in MS medium containing 2.0 mg L^-1^ 2,4-D. Scale bars, 1 cm. B) Hygromycin (Hyg)-resistant calli (bright yellow) formed after 8 weeks of selection on MS medium containing 2.0 mg L^-1^ 2,4-D, 100 mg L^-1^ cefotaxime sodium, and 11.0 mg L^-1^ Hyg. Scale bars, 1 cm. C) Regeneration of plantlets from Hyg-resistant calli on MS medium with 1.0 mg L^-1^ ABA and 100 mg L^-1^ cefotaxime sodium. Scale bars, 1 cm. D) Transgenic plant cultured on MS medium. Scale bars, 1 cm. E and F) PCR analysis of positive transgenic plants. Lane M: BL2000 DNA markers; Lane W: water as a negative control; Lane P: pCAMBIA1300-*IbCHYR1* or pFGC5941-*IbCHYR1* as a positive control; Lane WT: WT as a negative control. G) Transcript levels of *IbCHYR1* in transgenic and WT plants. The results are expressed as relative values with respect to the transcript level of the WT, which was set to 1.0. The values were determined by RT-qPCR from three biological replicates consisting of pools of five plants. The error Scale bars indicate ± Sd (*n* = 3). (**) *P* < 0.01; Student’s *t*-test.

**
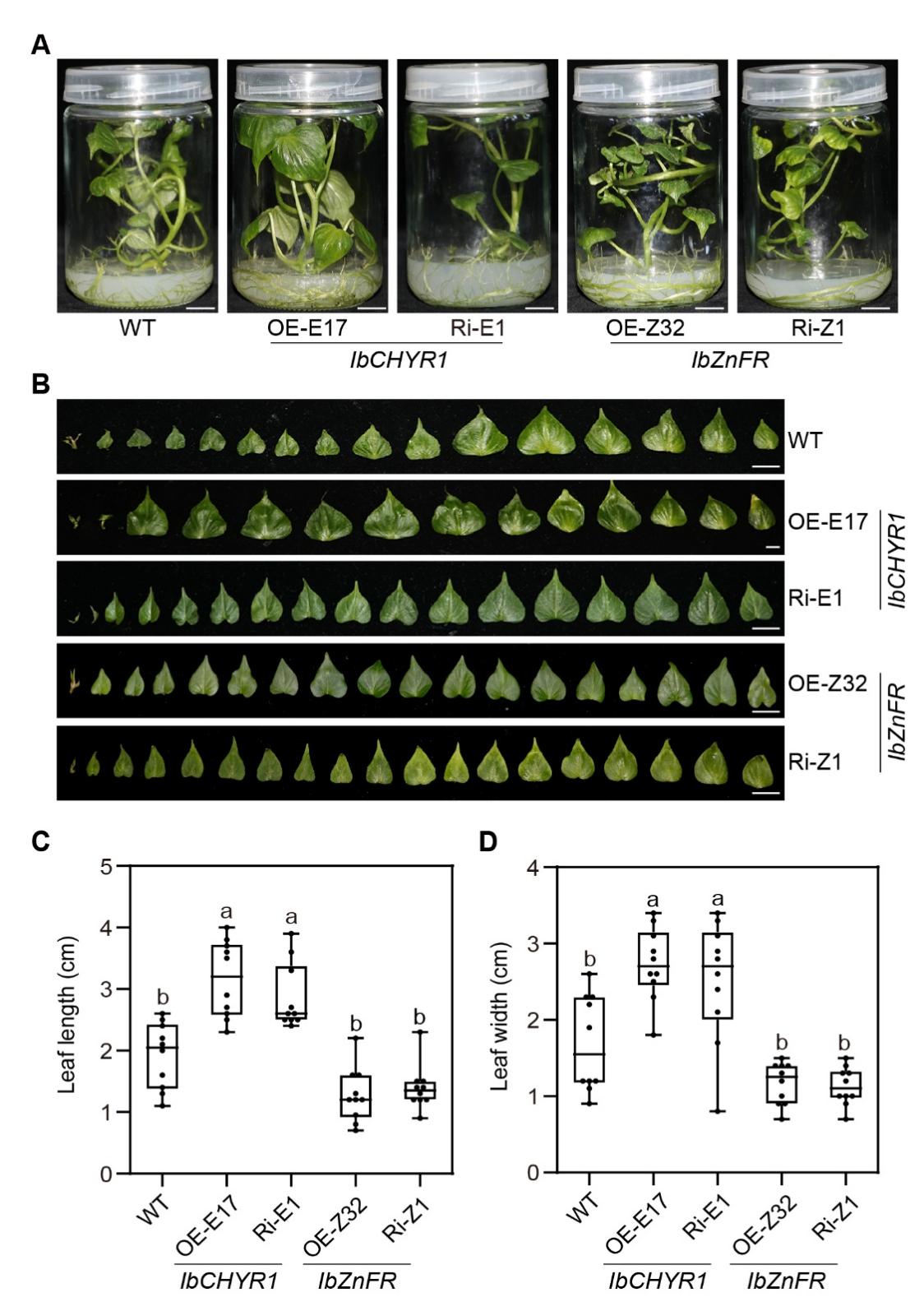
**

**Figure S7. *IbCHYR1* and *IbZnFR* regulate *in vitro*-grown plant development in sweet potato.** A) Plant morphology of 1-month-old *IbCHYR1* transgenic plants, *IbZnFR* transgenic plants, and WT plants. Scale bars, 2 cm. B) Changes in leaf morphology in *IbCHYR1* transgenic plants, *IbZnFR* transgenic plants, and WT plants in (A), arranged from the first leaf to the last leaf. Scale bars, 1 cm. C and D) Leaf length and width in *IbCHYR1* transgenic plants, *IbZnFR* transgenic plants, and WT plants. The error Scale bars indicate ± Sd (*n* = 10). Different letters indicate statistically significant differences (one-way ANOVA followed by a *post-hoc* Tukey test; *P* < 0.05).


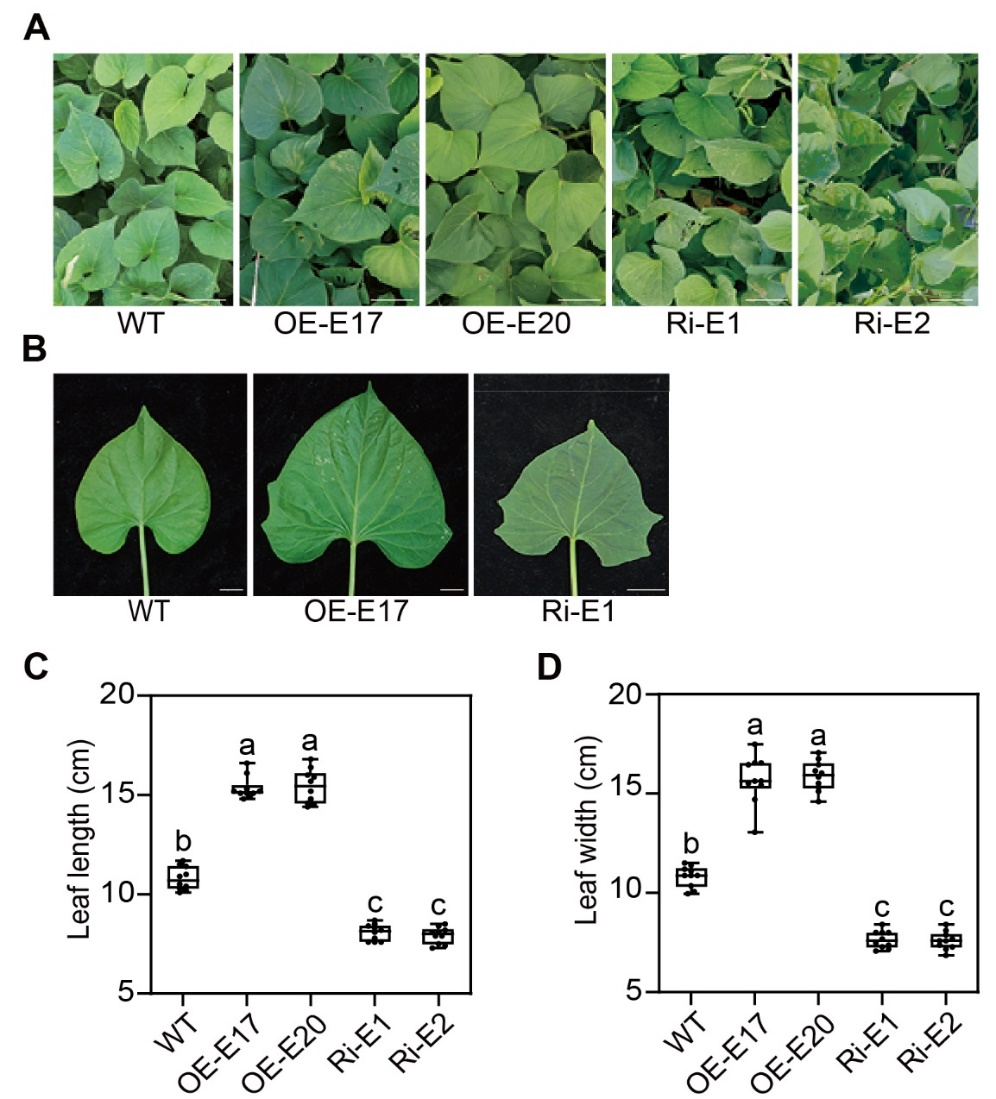


**Figure S8. *IbCHYR1* regulates field-grown plant development in sweet potato.** A) Morphology of 3-month-old *IbCHYR1* transgenic plants and WT plants. Scale bars, 7.5 cm. B) Changes in leaf morphology in *IbCHYR1* transgenic plants and WT plants in (A). Scale bars, 5 cm. C and D) Leaf length and width of *IbCHYR1* transgenic plants and WT plants. The error Scale bars indicate ± Sd (*n* = 10). Different letters indicate statistically significant differences (one-way ANOVA followed by a *post-hoc* Tukey test; *P* < 0.05).

**
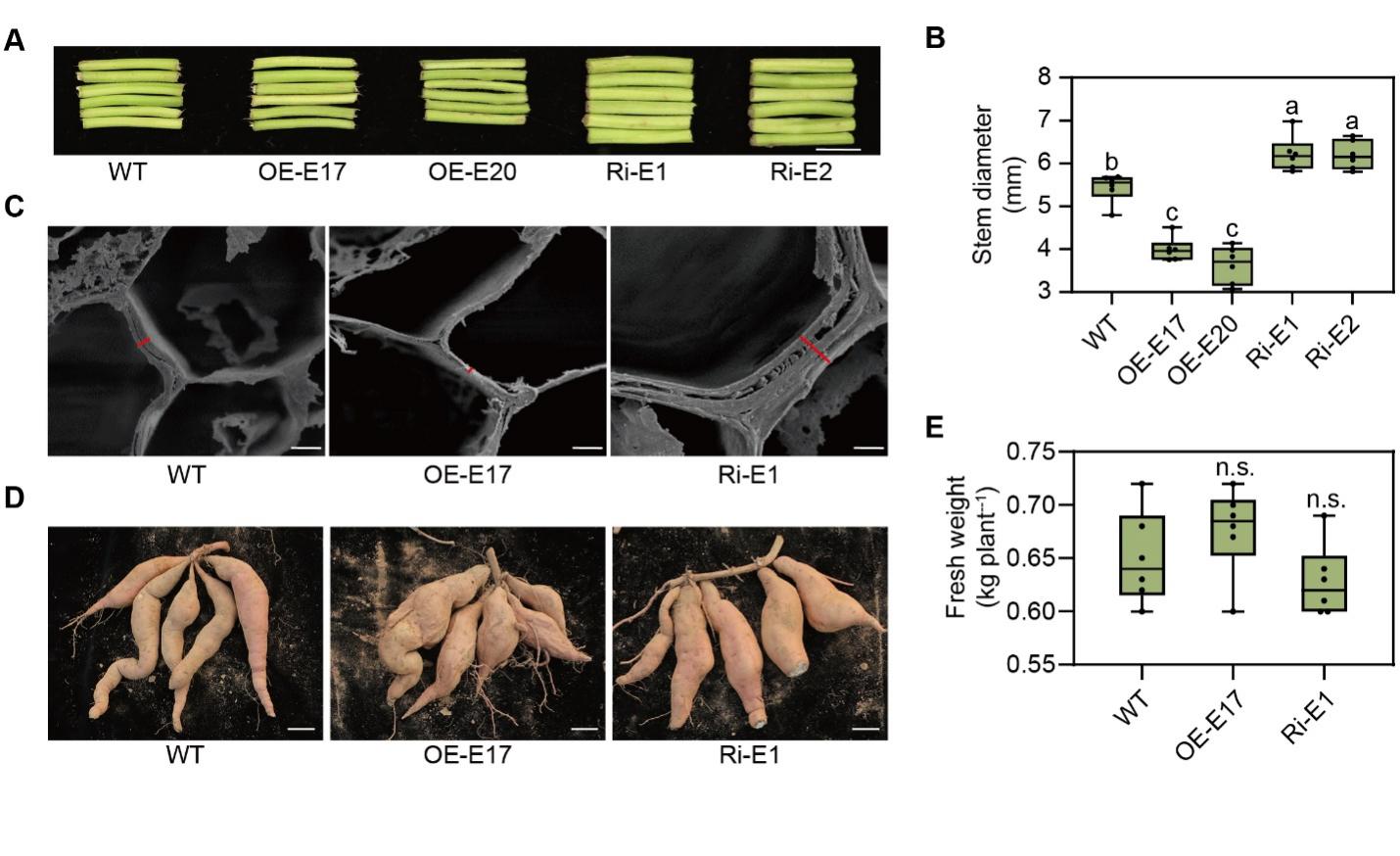
**

**Figure S9.** **Stem thickness and yield statistics of *IbCHYR1* transgenic plants.** A and B) Three-month-old field-grown *IbCHYR1*-OE plants had significantly thinner stems compared to the WT, in contrast to *IbCHYR1*-Ri plants. Scale bars, 1 cm. Data are shown as the means ± SD (*n* = 6). C) Scanning electron micrograph of a transversal section showing the cell wall thickness in *IbCHYR1* transgenic plants. Scale bars, 3 μm. D and E) Fresh weight of the storage roots of *IbCHYR1* transgenic and WT plants. Scale bars, 5 cm. Data are shown as the means ± SD (*n* = 6). In B) and E) different letters indicate statistically significant differences (one-way ANOVA followed by a *post-hoc* Tukey test; *P* < 0.05); n.s., not significant.


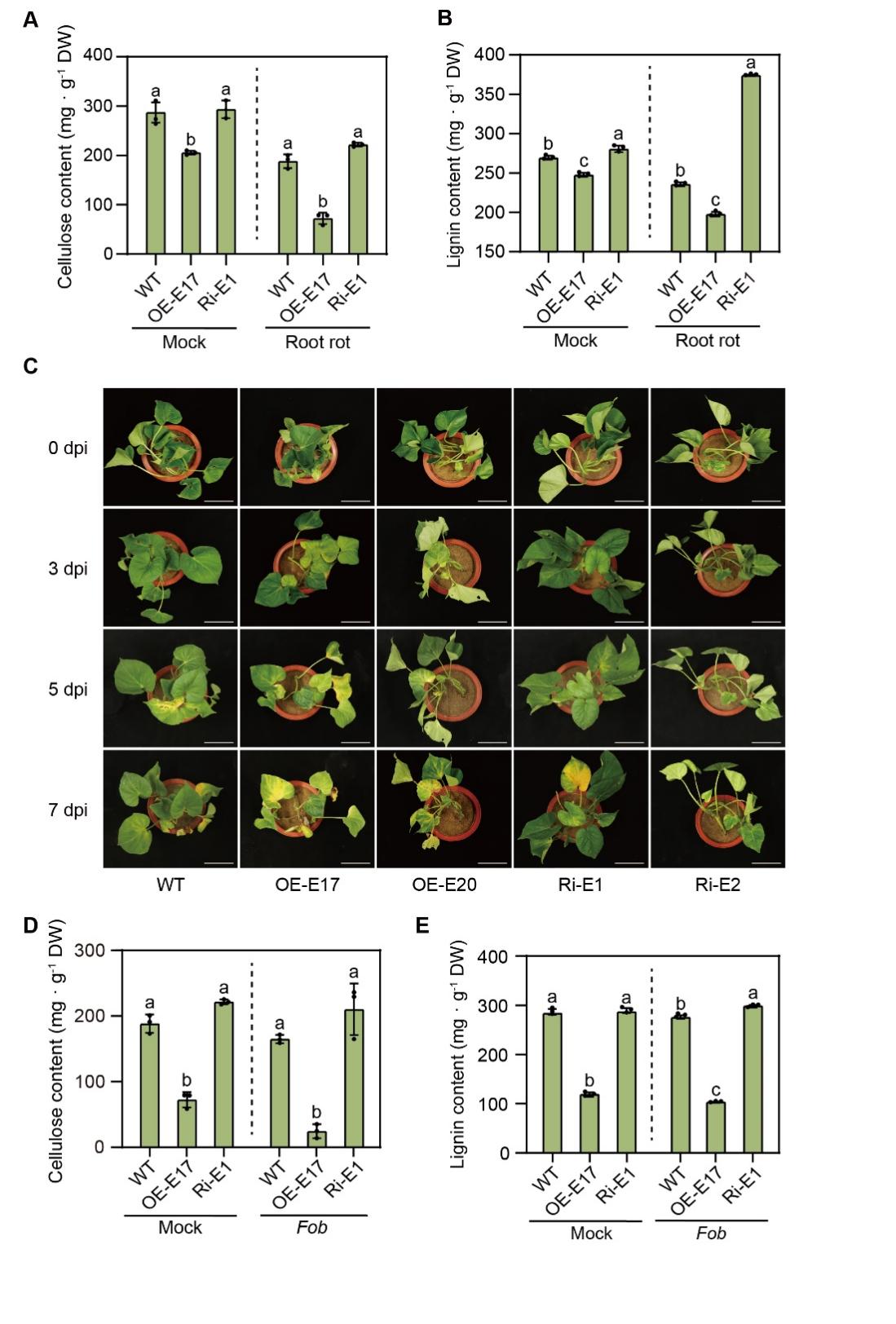


**Figure S10. *IbCHYR1* represses sweet potato resistance to root rot and *Fusarium* wilt.** A and B) Cellulose and lignin accumulation in the roots of *IbCHYR1* transgenic and WT plants in the root rot field. Data are shown as the means ± SD (*n* = 3). C) Development of disease symptoms in *IbCHYR1* transgenic and WT plants after *Fob* inoculation by spore. D and E) Cellulose and lignin accumulation in the stems of *IbCHYR1* transgenic plants and WT plants at 0 DAI and 1 DAI by the spore infection method. Data are shown as the means ± SD (*n* = 15). In (A), (B), (D), and (E), different letters indicate statistically significant differences (one-way ANOVA followed by a *post-hoc* Tukey test; *P* < 0.05). DW, dry weight.

**
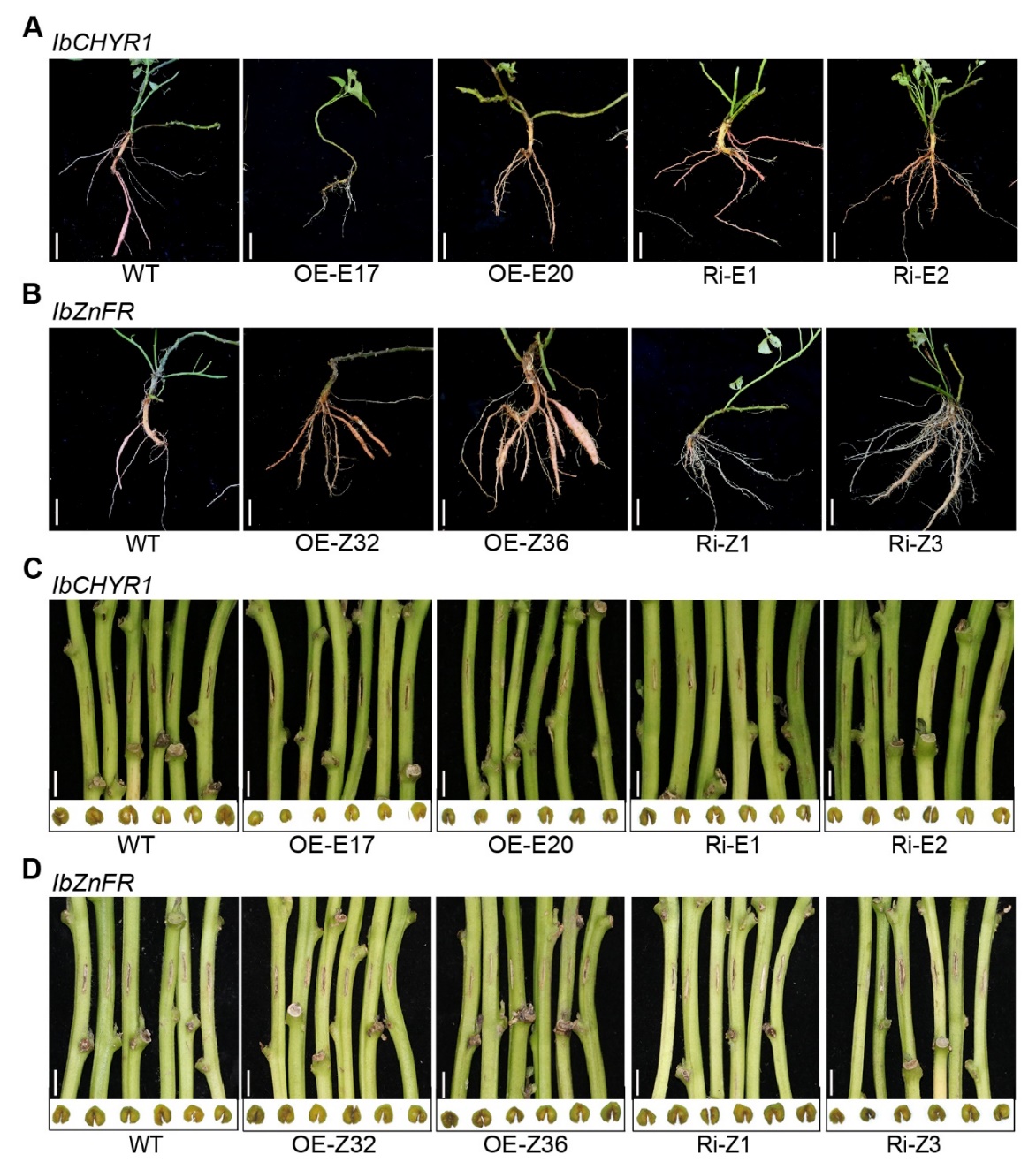
**

**Figure S11. Mock treatments for root rot and *Fusarium* wilt** **resistance assays.** A and B) Mock treatments performed for the root rot resistance assay. Plants were grown in a normal field for 45 days. Scale bars represent 4 cm. C and D) Mock treatments for the mycelia infection method for the *Fusarium* wilt resistance assay. Sterile potato dextrose agar tablets were placed on a 1-cm-long wound on the stems of plants. The images were taken at 15 days after planting. Scale bars represent 4 cm.


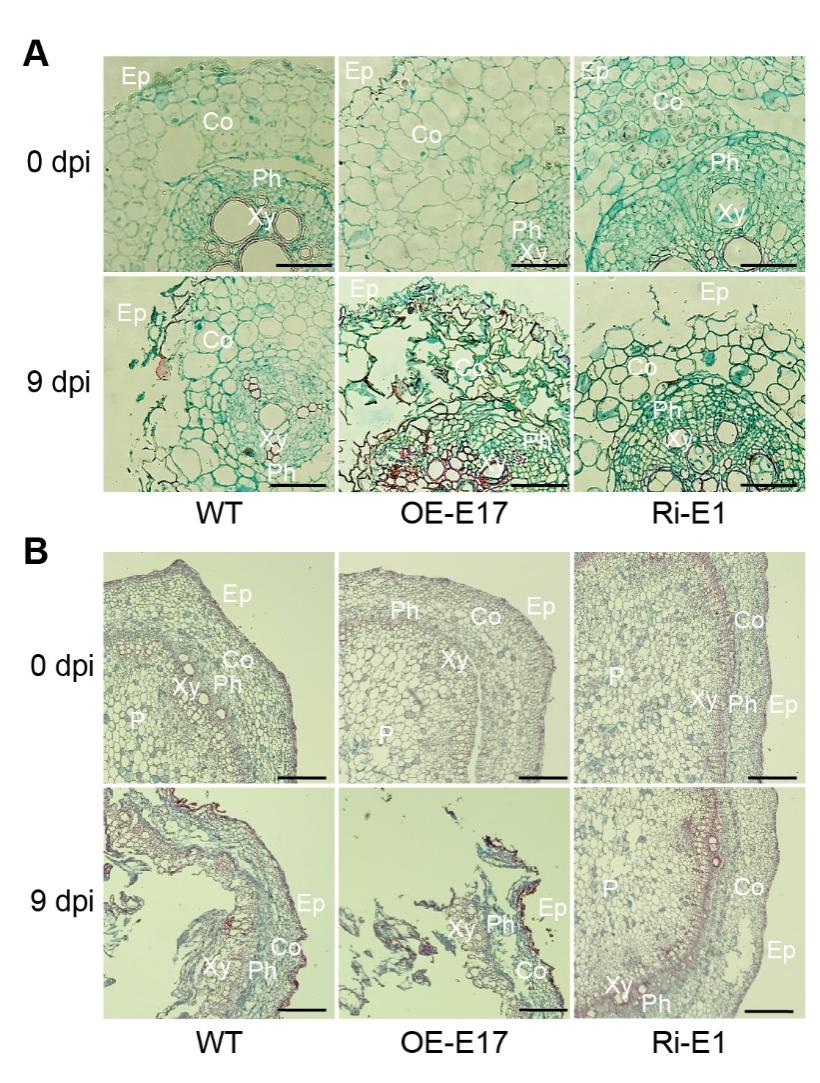


**Figure S12. Histological examination of *IbCHYR1* transgenic and WT plants.** A) Histological examination of root transverse sections of *IbCHYR1* transgenic plants and WT plants in a root rot field 0 and 9 DAP. Scale bars, 60 μm. B) Histological examination of stem transverse sections of *IbCHYR1* transgenic plants and WT plants infected with *Fob*. Scale bars, 120 μm. Co, cortex; Ep, epidermis; P, pith; Ph, phloem, Xy, xylem.

**
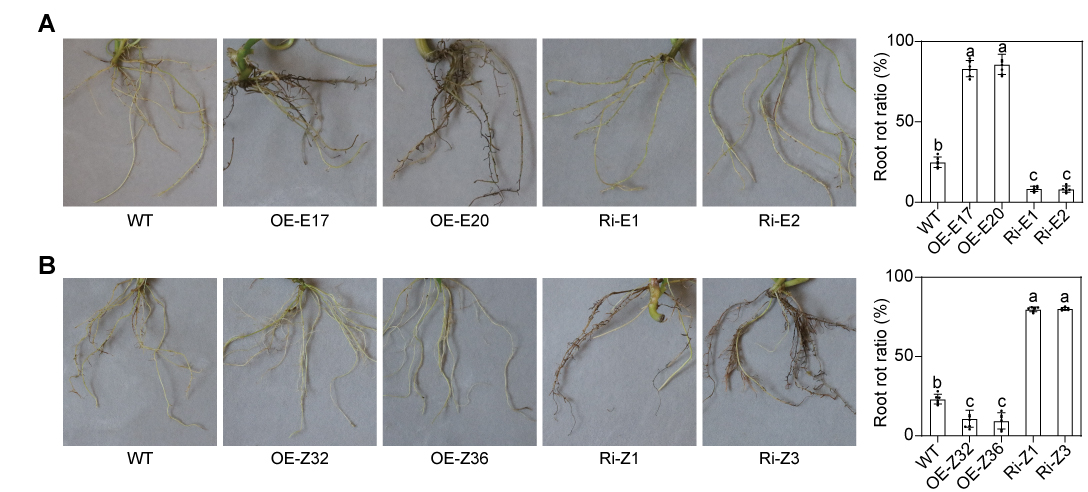
**

**Figure S13.** **DX94 infection in *IbCHYR1* transgenic plants (A) and *IbZnFR*** **transgenic plants (B).** Data are shown as the means ± SD (*n* = 6). Different letters indicate statistically significant differences (one-way ANOVA followed by a *post-hoc* Tukey test; *P* < 0.05).

**
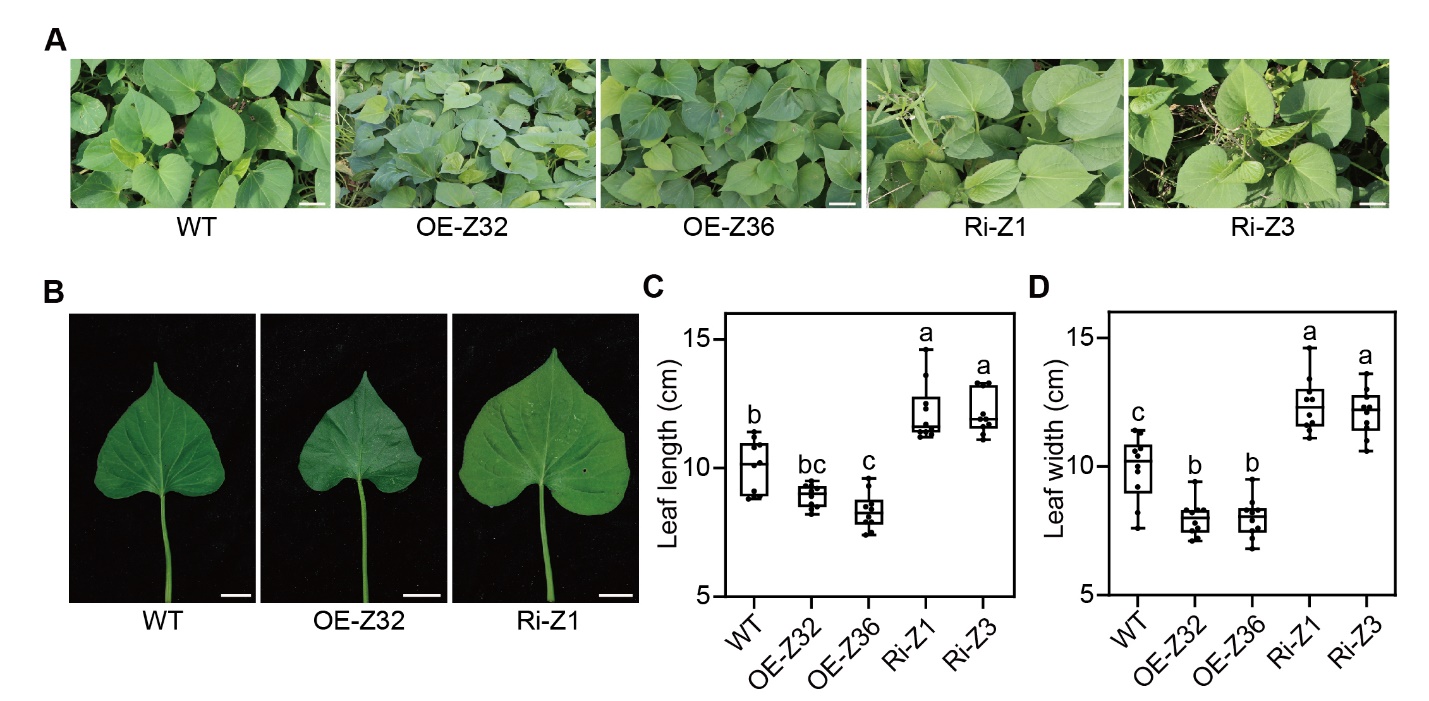
**

**Figure S14. *IbZnFR* regulates field-grown plant development in sweet potato.** A) Morphology of 3-month-old *IbZnFR* transgenic plants and WT plants. Scale bars, 5 cm. B) Changes in leaf morphology in *IbZnFR* transgenic plants and WT plants in (A). Scale bars, 5 cm. C and D) Leaf length and width of *IbZnFR* transgenic plants and WT plants. The error bars indicate ± S.D. (*n* = 10). Different letters indicate statistically significant differences (one-way ANOVA followed by a *post-hoc* Tukey test; *P* <0.05).


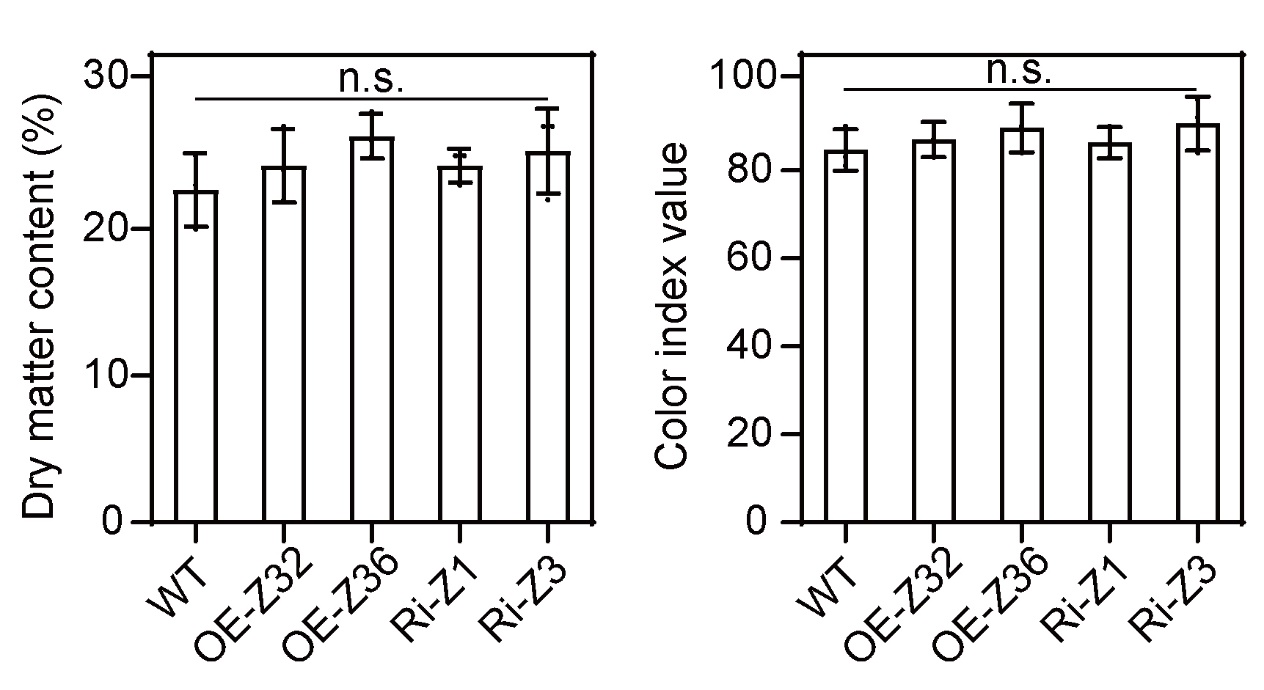


**Figure S15. D****ry matter content and color index values of the storage roots of *IbZnFR* transgenic and WT** **plants.** The error bars indicate ± S.D. (*n* = 3). n.s. indicate no statistically significant differences (one-way ANOVA followed by a post-hoc Tukey test; P <0.05). The color index values were measured using a Minolta Chromameter.


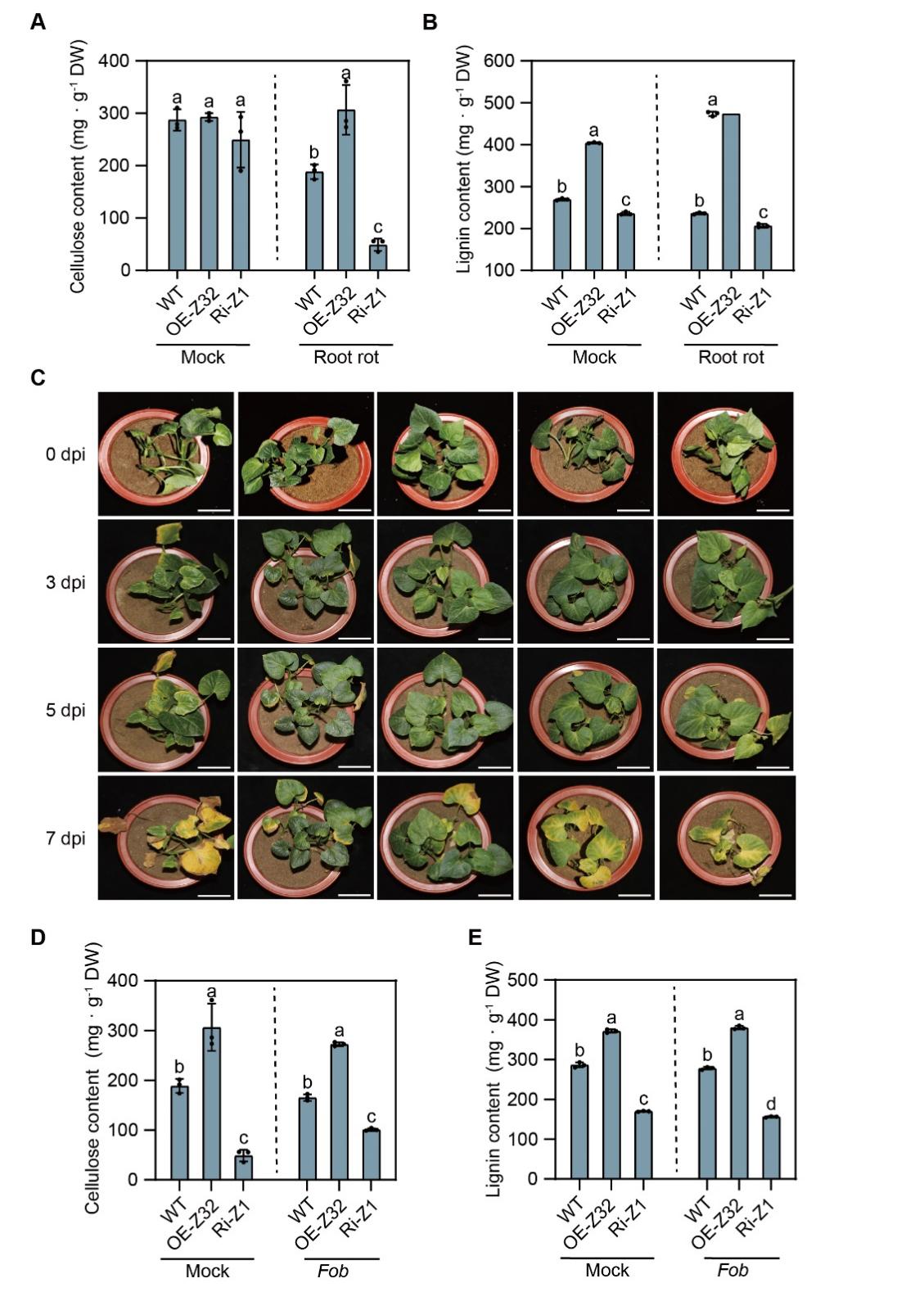


**Figure S16. *IbZnFR* enhances sweet potato resistance to root rot and *Fusarium* wilt.** A and B) Cellulose and lignin accumulation in the roots of *IbZnFR* transgenic and WT plants in the root rot field. Data are shown as the means ± SD (*n* = 3). C) Development of disease symptoms in *IbZnFR* transgenic and WT plants after *Fob* inoculation by spore. D and E) Cellulose and lignin accumulation in the stems of *IbZnFR* transgenic plants and WT plants at 0 DAI and 1 DAI by the spore infection method. Data are shown as the means ± S.D. (*n* = 15). In (A), (B), (D), and (E), Different letters indicate statistically significant differences (one-way ANOVA followed by a *post-hoc* Tukey test; *P* <0.05). DW, dry weight.


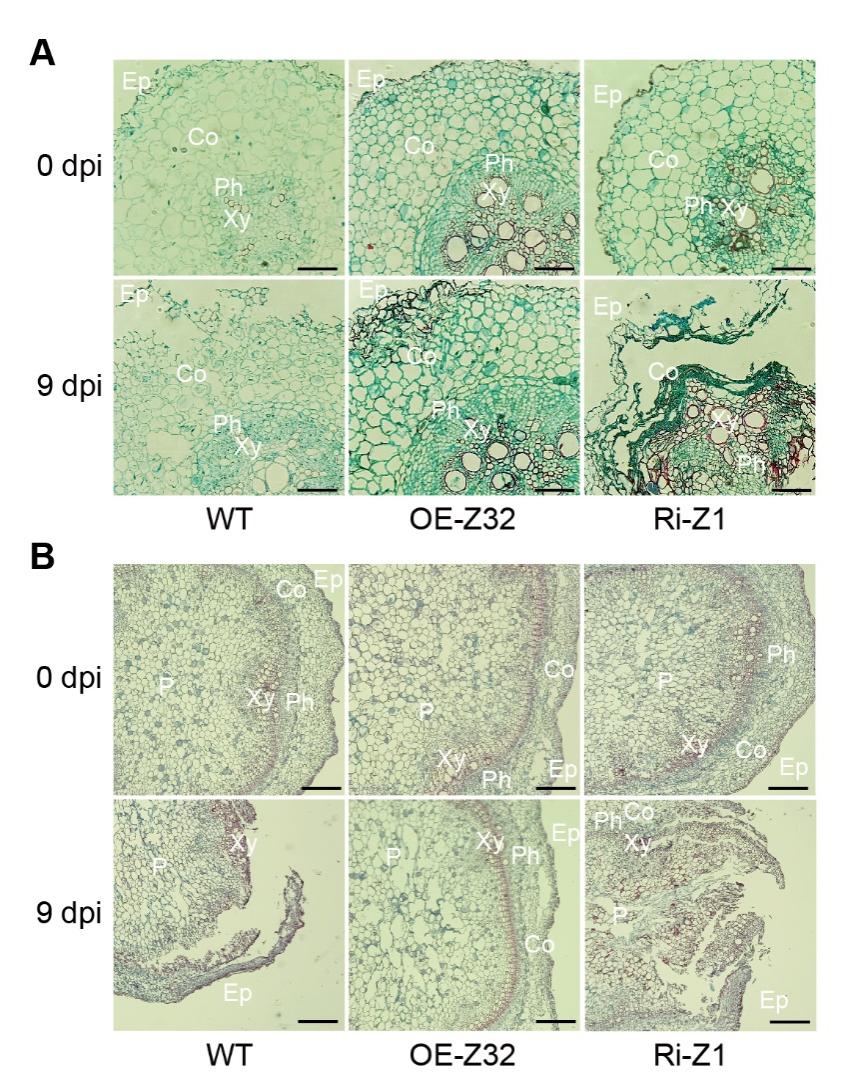


**Figure S17. Histological examination of *IbZnFR* transgenic and WT plants.** A) Histological examination of root transverse sections of *IbZnFR* transgenic plants and WT plants in a root rot field 0 and 9 DAP. Scale bars, 60 μm. B) Histological examination of stem transverse sections of *IbZnFR* transgenic plants and WT plants infected with *Fob*. Scale bars, 120 μm. Co, cortex; Ep, epidermis; P, pith; Ph, phloem, Xy, xylem.


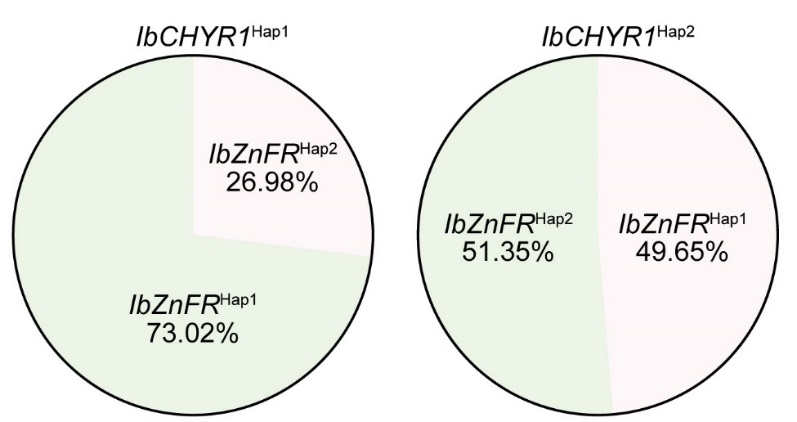


**Figure S18.** **The correlation between the distribution of *IbCHYR1* and *IbZnFR* haplotypes** **in 157 sweet potato accessions.**


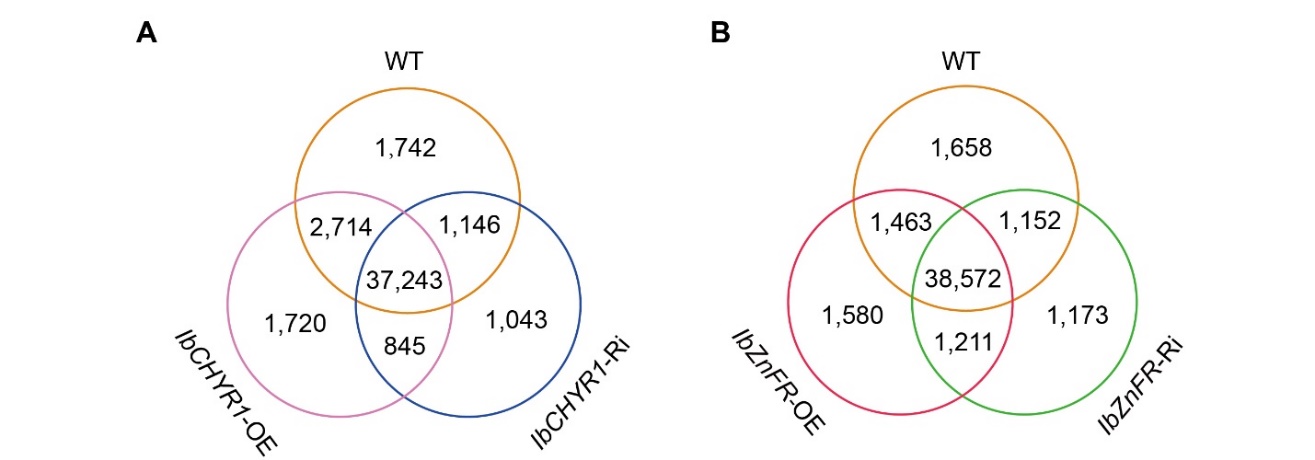


**Figure S19. Venn diagram showing the number of expressed genes of *IbCHYR1*** (**A**) **and *IbZnFR*** (**B**) **transgenic and WT plants 10 DAP in a root rot field.** A total of 48,358 genes were expressed across the *IbCHYR1* and *IbZnFR* transgenic plants and WT plants.

**
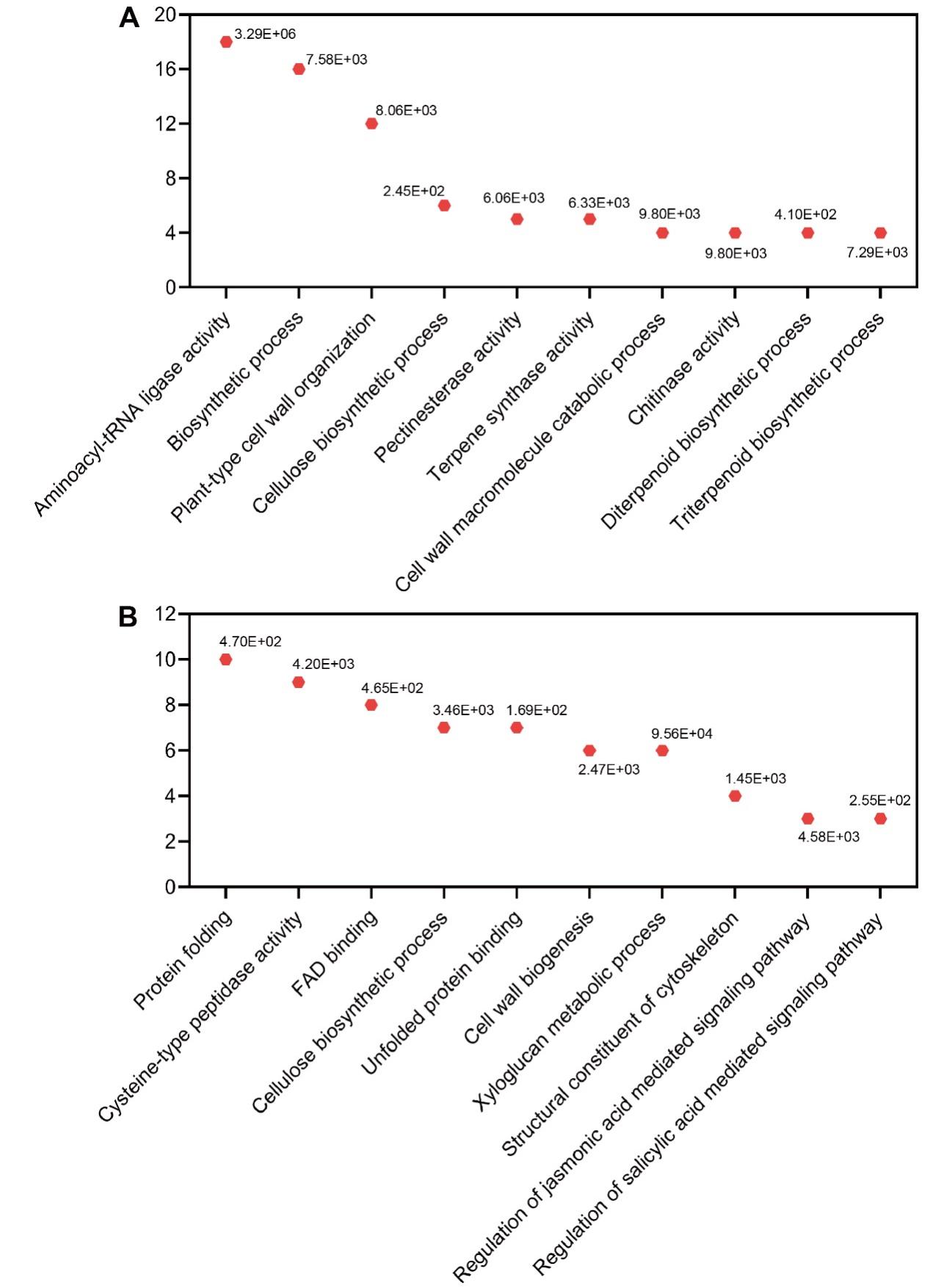
**

**Figure S20. GO enrichment of DEGs based on RNA-seq data for *IbCHYR1* and** ***IbZnFR*** **transgenic plants and WT plants 10** **DAP in a root rot field.** In total, 2,223 and 1,373 DEGs showing opposite expression patterns in the *IbCHYR1* (A) and *IbZnFR* (B) transgenic plants, respectively, were functionally annotated and classified using the GO database.


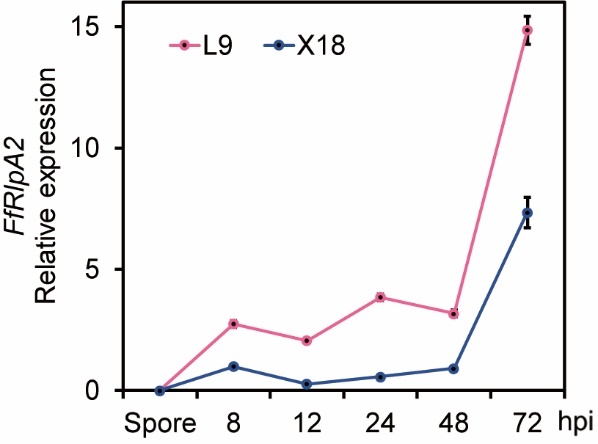


**Figure S21. RT-qPCR showed that *FfRlpA2* was highly induced in L9 after DX94 infection, compared to X18.** The DX94 *Actin* gene (*FfActin*, S5G05520) was used as an internal control. Three biological replicates consisting of pools of five plants were used for RT-qPCR. The error bars indicate ± SD (n = 3).


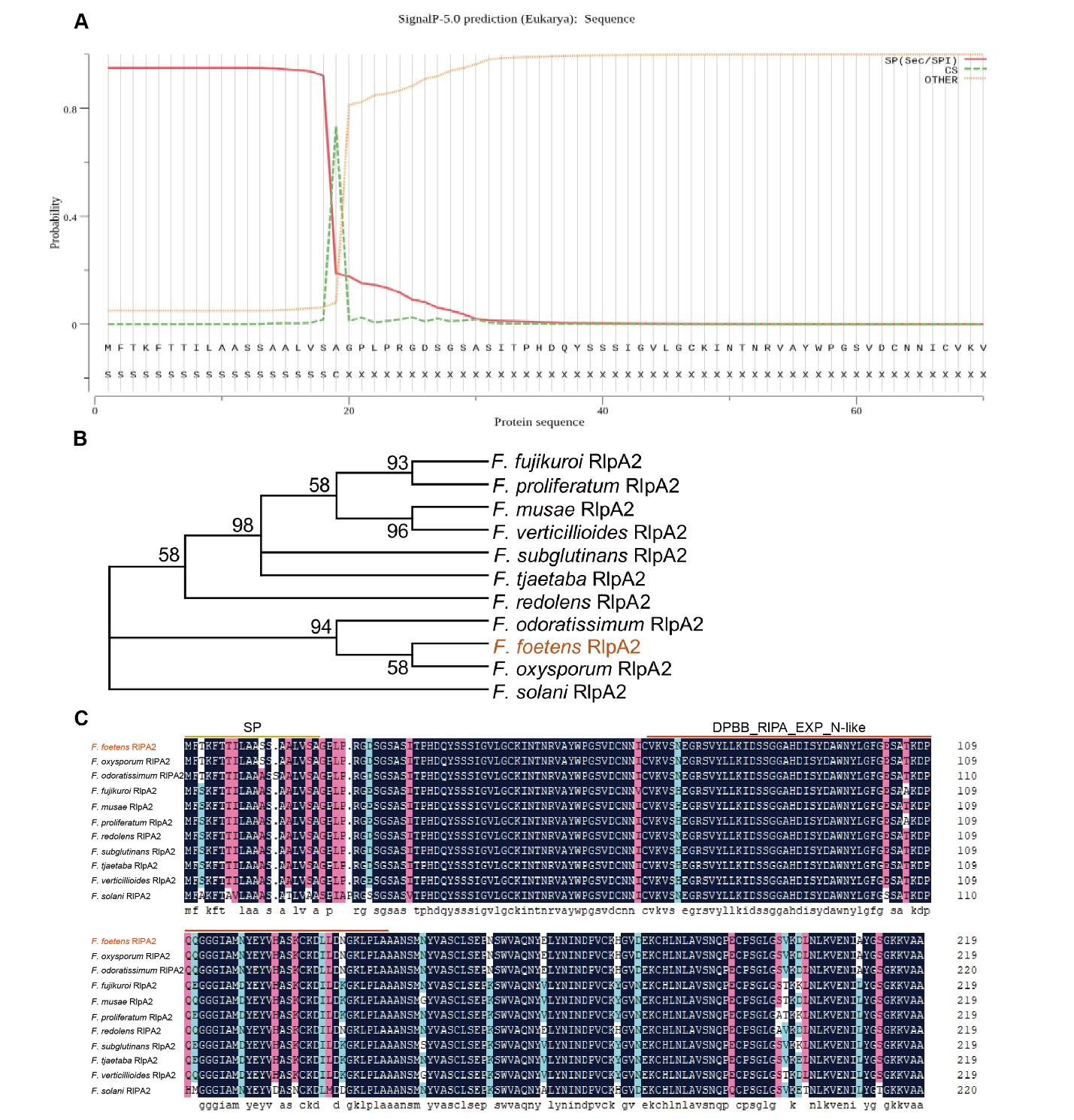


**Figure S22. The signal peptide (SP) analysis and phylogenetic analysis of FfRlpA2.** A) SP analysis of FfRlpA2. The red line shows the secretion SP in the first 19 amino acids. B) Phylogenetic analysis of RlpA2 proteins performed using the neighbor-joining method in MEGA6.0 with 1,000 bootstrap iterations. The numbers at the nodes of the tree indicate bootstrap values from 1,000 replicates. C) Multiple protein sequence alignment of *Ff*RlpA2 with other *Fusarium* RlpA2 proteins, with conserved amino acids shaded in different colors. The double-psi beta-barrel (DPBB)_RlpA_expansin (EXP)_N-like domain is outlined.


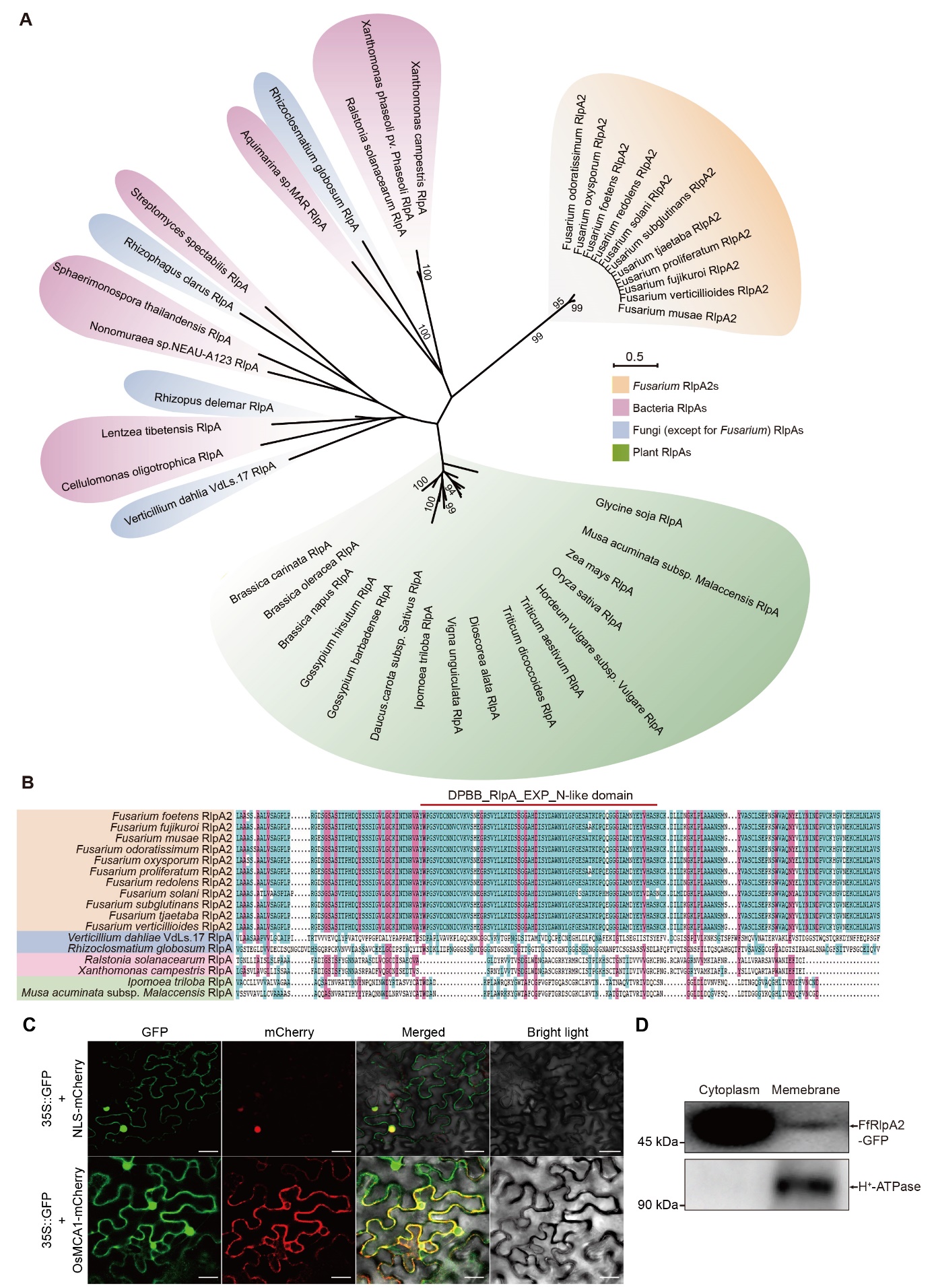


**Figure S23.** **Phylogenetic analysis of RlpA proteins in plants, fungi, and bacteria.** A) Phylogenetic analysis of RlpA proteins performed using the neighbor-joining method in MEGA6.0 with 1,000 bootstrap iterations. The numbers at the nodes of the tree indicate the bootstrap values of 1,000 replicates. Background colors represent distinct species: brown represents *Fusarium*; pink represents bacteria; blue represents fungi (except for *Fusarium*); and green represents plants. B) Multiple protein sequence alignment of RlpA proteins (9–197 aa), with conserved amino acids shaded in different colors. The double-psi beta-barrel (DPBB)_RlpA_expansin (EXP)_N-like domains are outlined. C) The pCAMBIA1300-GFP vector was used as a native control of FfRlpA2 in Figure 6H. The pCAMBIA1300-GFP was co-expressed with the nuclear marker NLS-mCherry or the plasma membrane marker OsMCA1-mCherry. Scale bars, 20 μm. D) Subcellular fractionation assays of FfRlpA2. H⁺-ATPase was used as a membrane-localized positive control.


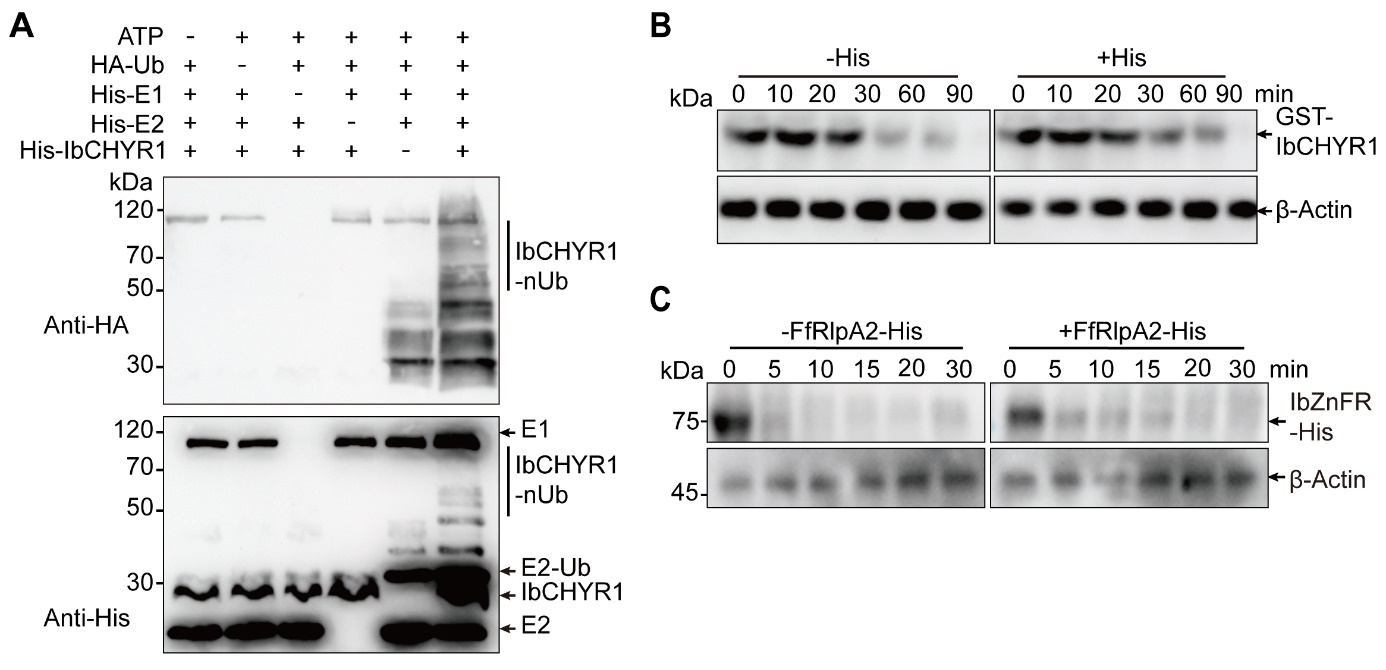


**Figure S24. IbCHYR1 possesses auto-ubiquitination activity.** A) E3 ubiquitin ligase activity assay showing that the IbCHYR1 protein had auto-ubiquitination activity in the presence of E1 and E2. B) Recombinant GST-IbZnFR and His proteins were incubated with a crude protein, which was used as a native control of Figure 7B. C) Recombinant IbZnFR-His and FfRlpA2-His proteins were incubated with a crude protein, which was used as a native control of Figure 7F. Aliquots of the mixture were collected at the indicated times and probed by immunoblotting with anti-His antibody, β-Actin was used as a loading control.

**Table S1. Sequences of the primers and probes used in this study.**

**Table S2.** **446 SNPs in the LD blocks of the significant loci (Iba_chr13a_11097067 and Iba_chr14a_31763570) associated with root rot resistance were identified by GWAS.**

**Table S3. List of 283 DEGs upregulated in the roots of two susceptible varieties but downregulated in resistant varieties by RNA-seq at 10 DAP in a root rot field.**

**Table S4. The FPKM of the putative interacting partners of IbCHYR1 identified by yeast two-hybrid and IP-MS screening.**

**Table S5.** **List of 709 and 1514 DEGs exhibiting opposite trends in expression patterns between *IbCHYR1*-OE plants and *IbCHYR1*-Ri plants, compared to the WT, 10 DAP in a root rot field.**

**Table S6.** **List of 781 and 592 DEGs exhibiting opposite trends in expression patterns between *IbZnFR*-OE plants and** ***IbZnFR*-Ri plants, compared to the WT, 10 DAP in a root rot field.**

**Table S7. List of DEGs exhibiting opposite expression patterns between *IbCHYR1* and *IbZnFR* transgenic plants, compared to the WT, 10 DAP in a root rot field.**

**Table S8.** **List of the overlapping DEGs detected by RNA-Seq and ChIP-Seq.**

**Table S9. A total of 686 DEGs including 14 effector genes downregulated in two resistant varieties but upregulated in two susceptible varieties.**

**Table S10.** **List of 61 overlapping DEGs in *IbCHYR1* and *IbZnFR* transgenic plants, 10 DAP in a root rot field, in the DX94 genome. Effectors are labeled in red.**
